# Reactive cholangiocyte-derived ORM2 drives a pathogenic modulation of the injured biliary niche through macrophage reprogramming

**DOI:** 10.1136/gutjnl-2024-334425

**Published:** 2025-04-08

**Authors:** Hanyang Liu, Guo Yin, Bianca Franco Leonardi, Tian Lan, Yeni Ait Ahmed, Hilmar Berger, Marlene Sophia Kohlhepp, Natalja Amiridze, Natalia Martagón Calderón, Carla Frau, Ludovic Vallier, Milad Rezvani, Frank Tacke, Adrien Guillot

**Affiliations:** 1 Department of Hepatology and Gastroenterology, Campus Virchow-Klinikum and Campus Charité Mitte, Charité - Universitätsmedizin Berlin, Berlin, BE, Germany; 2 Cell Biology and Imaging Section, Thoracic and GI Malignancies Branch, Center for Cancer Research, National Cancer Institute, National Institutes of Health, Bethesda, Maryland, USA; 3 Department of Physiology and Biophysics, Institute of Biomedical Sciences, University of Sao Paulo, Sao Paulo, Brazil; 4 Laboratory of Gastroenterology and Hepatology, State Key Laboratory of Biotherapy, West China Hospital, Sichuan University, Chengdu, China; 5 Department of Pediatric Gastroenterology, Nephrology and Metabolic Medicine, Charité – Universitätsmedizin Berlin, corporate member of Freie Universität Berlin and Humboldt Universität zu Berlin, Berlin, Germany; 6 BIH Center for Regenerative Therapies (BCRT), Berlin, Germany, Berlin Institute of Health (BIH) at Charité-Universitätsmedizin Berlin, Berlin, Germany; 7 Max-Planck-Institute for Molecular Genetics, Berlin, Germany

**Keywords:** BILIARY EPITHELIUM, CHOLESTATIC LIVER DISEASES, LIVER IMMUNOLOGY, MACROPHAGES, BILIARY PHYSIOLOGY

## Abstract

**Background:**

Injured or reactive biliary epithelial cells participate in most chronic liver injuries in a process referred to as ductular reaction, which involves multicellular interactions with marked local infiltration of macrophages and fibrogenic cell activation. The direct roles of biliary epithelial cells in shaping their cellular niche remain unknown.

**Objective:**

We aimed at investigating the effects of biliary epithelial cell-derived acute phase response protein orosomucoid 2 (ORM2) in shaping monocyte/macrophage response to liver injury.

**Design:**

Transcriptome data sets from human and mouse livers were used, results were confirmed with multiplex immunofluorescence. A multicellular biliary-niche-on-a-chip derived from primary liver and blood cells (wild-type, *Mdr2*^−/−^ mice) was established to model ductular reaction. Human blood cells collected from healthy donors and intrahepatic cholangiocyte organoids derived from normal and cirrhotic liver patients were used.

**Results:**

Our transcriptome data set and multiplex immunofluorescence analyses indicated a previously unrecognised involvement of the acute phase response protein ORM2 in ductular reactions in both human and mouse livers. ORM2 gene expression was increased in biliatresone-challenged, bile acid-challenged and acetaminophen-challenged cholangiocytes. Cholangiocyte-derived ORM2 induced unique transcriptome changes and functional adaptation of liver macrophages. ORM2-activated macrophages exacerbated cholangiocyte cell stress and *Orm2* expression, but also tended to promote fibrogenic activation of hepatic stellate cells. Mechanistically, ORM2 effects were mediated by an inositol 1,4,5-trisphosphate receptor type 2-dependent calcium pathway.

**Conclusion:**

This study reveals a paracrine communication circuit during ductular reaction, in which reactive cholangiocyte-derived ORM2 reprogrammes liver macrophages, participating in a pathogenic remodelling of the immune biliary niche.

**WHAT IS ALREADY KNOWN ON THIS TOPIC**

- Intrahepatic reactive biliary epithelial cell accumulation (i.e., ductular reaction) is a hallmark of liver disease progression, yet significant gaps remain in understanding its functional impact on disease course.

**WHAT THIS STUDY ADDS**

- Our study reveals that reactive biliary epithelial cell-derived orosomucoid 2 reprogrammes liver macrophages for a pathogenic remodelling of the immune biliary niche, shedding new light on intricate cellular interactions during liver injury.

**HOW THIS STUDY MIGHT AFFECT RESEARCH, PRACTICE OR POLICY**

- These findings emphasise the overlooked potential of ductular cells and their cellular cross-talk as a therapeutic target for treating liver diseases.

## Introduction

 Epithelial cell injury is a hallmark of liver diseases, which creates an inflammatory environment, potentially allowing regenerative processes by hepatocyte-cholangiocyte progenitors marked by epithelial plasticity.^1^ Despite different injury triggers in, for instance, cholestatic or metabolic dysfunction-associated steatotic liver diseases (MASLD), chronic injury, fibrogenic responses and regenerative mechanisms culminate in the so-called ductular reaction, which is classically characterised by the expansion of biliary epithelial cell (BEC)-derived ductular cells. This phenomenon is occurring in conjunction with the accumulation of inflammatory cells, mainly monocyte-derived macrophages (MoMF) and proliferative mesenchymal and vascular cells.^2^ In our prior work, the accumulation of IBA1^+^ MoMF and their proximity to CK19^+^ ductular cells was closely associated with the severity of fibrosis in human livers from individuals with MASLD or cholangiopathies such as primary sclerosing cholangitis (PSC).^3^ Despite this well-known association between ductular reaction, inflammation and fibrosis, the molecular mechanisms underlying cellular crosstalk in ductular reaction and determining the outcome, that is, fibrosis versus repair, are incompletely understood.^2^

In conditions of chronic liver injuries, cholangiocytes adopt a reactive and secretory phenotype leading to the release of soluble mediators termed cholangiokines.^4^ These mediators induce pathogenic responses in neighbouring cells, as ductular cell depletion reduces liver fibrosis and inflammation in *Mdr2*^−/−^ mice, a model of chronic cholestatic injury.^5^ As such, it is increasingly recognised that ductular cells represent more than just bystanders in liver disease progression.^6^ For instance, acutely injured BECs express the chemokine (C-C motif) ligand 2 (CCL2), thus directly promoting local monocyte recruitment and, consequently, fostering local inflammation that drives either fibrosis progression or tissue repair.^7 8^ Indeed, diverse liver macrophage populations coexist, and each of them may adopt a plethora of activation phenotypes with a broad range of functions.^9^ Bile duct-associated macrophages have been shown to express markers classically associated with lipid-associated macrophages [e.g., triggering receptor expressed on myeloid cells 2 (TREM2), osteopontin].^7 9 10^ In mouse models of portal area injury, C-C chemokine receptor type 2 (CCR2)-positive monocyte-derived macrophages promote disease progression.^11^ However, much remains unknown about the direct influence of reactive cholangiocytes on the phenotypic peculiarities of bile duct-associated liver macrophages, and the impact on the course of liver disease.

Acute phase proteins (APPs), which are mainly produced in hepatocytes, regulate hepatic and systemic inflammation via mediating tissue repair, cellular metabolism and innate immune responses during injury.^12 13^ As a member of the APP family, increasing orosomucoid 2 (ORM2) concentration is known to increase in serum of individuals with infections and ongoing inflammation.^14^ ORM2 can regulate innate immune responses, cellular ion transportation and bile acid metabolism.^15^,^18^ In addition, a recent study emphasised that ORM2 directly promotes macrophage-driven inflammation, thereby aggravating chronic arthritis in mice.^19^ Besides, ORM2 was revealed to improve hepatic steatosis by enhancing hepatocellular lipid metabolism and regulating liver inflammation via the calcium pathway,^20^ while in another context, ORM2 was determined to decrease lipid absorption, mediate overall lipid metabolism and obesity progression.^15 21 22^ Nevertheless, those studies remain elusive on the source of ORM2 and mostly report on the effects of ORM2 on hepatocytes and not on cholangiocytes.

In this study, we identified ORM2 as a key candidate regulator of pathogenic cholangiocyte-macrophage crosstalk on biliary injury. Altogether, we provided mechanistic evidence that cholangiocyte-derived ORM2 drives a pro-inflammatory phenotypic change in liver macrophages via regulating calcium signalling.

## Materials and methods

### Human samples

Archival human liver sections and patient-derived cholangiocyte organoids were used in this study. The donors were informed and consented to the use of their biological material in scientific research.

### Patient and public involvement

Patients and/or the public were not involved in the design, or conduct, or reporting, or dissemination plans of this research.

### Animals

C57BL/6J wild-type (WT) mice (18–24-week-old), reporter transgenic (actin-dsRed and actin-CFP on a B6 genetic background) and *Mdr2*^−/−^ mice (on a C57BL/6J genetic background) received an isoflurane overdose prior to primary liver and blood cell isolation. All animal procedures were approved by the State Office for Health and Social Affairs, Berlin (Registration number: T-CH 020/22, T-CH0028/24 and G 0243/19).

### Cell culture and in vitro experiments

Primary liver and blood cells were isolated from healthy mice. The perfusable liver-on-a-chip (LoC) with primary mouse cells has been described previously.^23^ Cholangiocytes were seeded in lieu of hepatocytes or added to the previously described LoC to obtain the biliary-niche-on-a-chip (BoC) (details are provided in online supplemental material). Human intrahepatic cholangiocyte organoids (hICOs) were generated from patients in the Charité-Universitätsmedizin Berlin with fibrosis stage-0 (F0) and stage-4 (F4), as previously described.^24^

### Statistical analysis

GraphPad Prism V.9.0 (GraphPad Software, USA), FlowJo (V.10.8.0, BD Biosciences, USA) and RStudio (V.2023.03.1 Build 446; plugins ‘ggplot2’) were used to generate graphs, plots and matrixes.^25^ Heatmaps represent relative values compared with the indicated control. The statistical tests used in this study are indicated in the figure legend for each panel. Data are presented as the mean±SD ‘p<0.05’ was considered to be significantly different.

Additional information is provided in the online supplemental materials.

## Results

### ORM2 is upregulated in injured BECs and associated with liver macrophage accumulation

**Figure 1:**
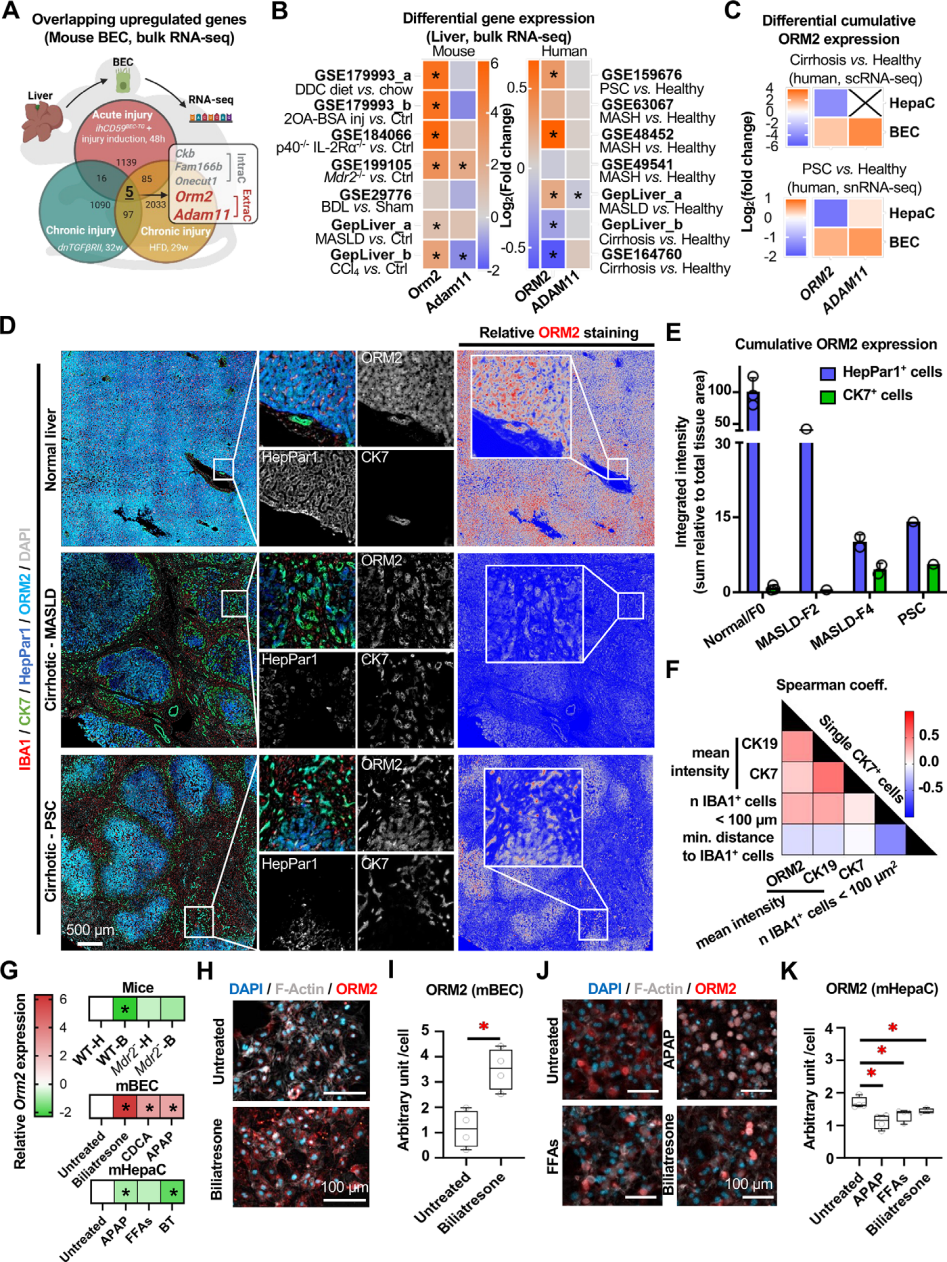
ORM2 is upregulated in cholangiocytes but downregulated in hepatocytes during liver injury. (**A**) *Ckb*, *Fam166b*, *Onecut1*, *Orm2* and *Adam11* were upregulated in both acutely (*ihCD59*^BEG-TG^ mice challenged by acute BEC injury for 48 hours, data set ID: PRJNA510784) and chronically (32-week-old *dnTGFβRII* mice, data set ID: GSE202676; 29-week HFD fed mice, data set ID: GSE217739) injured mouse BECs. Upregulated DEGs were identified as log_2_(fold change) >0.5, p<0.05. (**B**) Gene expression changes of *Adam11* and *Orm2* in injured livers from diverse mouse models (injured vs healthy) and human samples (diseases vs healthy). Bulk RNA sequencing data was obtained from the GepLiver database and GEO data sets. (**C**) Gene expression changes of *Orm2* in single-cell resolved human liver samples with MASLD, liver cirrhosis and PSC. scRNA-seq and snRNA-seq data were obtained from the GepLiver database and GEO data sets (ID: GSE247128 and GSE243977). ‘X’ in the block represents undetectable data. (**D**) Immunofluorescent staining illustrated the associations among hepatocytes (HepPar1^+^), BECs (CK7^+^), monocytes/macrophages (IBA1^+^) and ORM2 expression in human liver samples (normal, MALSD and PSC). Relative ORM2 staining was illustrated with highlighted portal areas. (**E**) ORM2 expression in single hepatocytes and BECs in human liver samples (normal, MALSD and PSC) was assessed according to the immunofluorescent staining analyses. (**F**) Significant correlations (Spearman’s, p<0.05) among staining intensity of ORM2, CK7 and CK19, neighbouring IBA1^+^ cells (<100 µm) and minimum distance to IBA1^+^ cells were illustrated in a heatmap. (**G**) Gene expression of *Orm2* in WT mouse primary BECs on biliatresone and CDCA treatments (vs untreated). (**H**) Protein expression of ORM2 in mouse primary BECs on biliatresone illustrated in fluorescent staining and (**I**) quantitative analysis. (**J**) Gene expression of *Orm2* in WT mouse primary hepatocytes on FFAs, APAP and biliatresone treatments (vs untreated). (**K**) Protein expression of ORM2 in mouse primary hepatocytes on biliatresone illustrated in fluorescent staining and (**L**) quantitative analysis. Sample sizes: (**D–F**) n=1–4 per group; (**G–K**) n=4 per group. APAP, acetaminophen; B/(m)BEC, (mouse) biliary epithelial cell; BSA, bovine serum albumin; CDCA, chenodeoxycholic acid; DDC, 3,5-diethoxycarbonyl-1,4-dihydrocollidine; DEG, differentially expressed gene; F0/2/4, fibrosis stage-0/2/4; FFAs, free fatty acids; H/(m)HepaC, (mouse) hepatocyte; HFD, high-fat diet; IntraC/ExtraC, intracellular/extracellular; MASLD, metabolic dysfunction associated steatotic liver diseases; MASH, metabolic dysfunction associated steatohepatitis; PSC, primary sclerosing cholangitis; OA, oleic acid; ORM2, orosomucoid 2; sc/snRNA, single-cell/single-nuclei RNA; WT, wild-type. One-way analysis of variance followed by Tukey’s multiple comparison test and unpaired Student’s t-tests were performed. *p<0.05 as indicated or as compared with controls.

To identify key factors consistently secreted by reactive BECs on ductular reaction in distinct settings, we retrieved bulk RNA sequencing (RNA-seq) data sets generated from purified BECs in acute (*ihCD59*^BEC-TG^ + bacterial toxin, 48 hours) and chronic (*dnTGFβRII*, 32 weeks) BEC injury mouse models or chronic (29 weeks) high-fat diet feeding as an MASLD model.^8 26 27^ Significantly upregulated differentially expressed genes (DEGs) were analysed from these three data sets (vs each internal control). *Ckb*, *Fam166b*, *Onecut1*, *Orm2* and *Adam11* were filtered out as co-upregulated genes using an overlapping analysis (figure 1A). While *Ckb*, *Fam166b* and *Onecut1* encode intracellular proteins, *Orm2* and *Adam11* encode surface or secreted extracellular proteins prone to serve as cell crosstalk mediators.^28 29^ Besides, publicly-available bulk RNA-seq data sets from multiple mouse models and patients with liver diseases^30^,^40^ indicated that *ORM2*/*Orm2* expression was more strongly upregulated than *ADAM11/Adam11* in cirrhotic and PSC livers (vs healthy control). However, *ORM2* expression was downregulated during liver cirrhosis in two independent data sets from human patients (vs healthy control) (figure 1B). Intriguingly, total (sum of individual cells) expression of *ORM2* tended to be downregulated in hepatocytes but upregulated in BECs, indicating that there may not be changes in gene expression at singular cell levels but rather a broader contribution of BECs to the overall *ORM2*/*Orm2* expression through the organ (cirrhosis, or PSC vs healthy control), which was not observed for *ADAM11* (figure 1C and online supplemental figure S1). Next, we conducted multiplex immunofluorescence staining from human liver illustrating that hepatocytes were the main contributors to tissue-wide ORM2 expression in healthy liver, while there appeared to be a shift towards an increased ORM2 signal originating from CK7-positive ductular cells on MASLD and PSC (figure 1D–E). Furthermore, we observed that CK7-positive cell ORM2 and CK19 expressions were positively correlated with a close vicinity from IBA1^+^ macrophages (figure 1F), indicating that ORM2 could be involved in the crosstalk between BECs and bile duct-associated macrophages. In line, a re-analysis of a previously published transcriptomic data set^20^ revealed that healthy mouse livers expressed higher levels of monocyte-associated markers after systemic ORM2 injection (online supplemental figure S2A–N).

To further interrogate the relationship between liver epithelial cell ORM2 expression and tissue injury, we treated primary mouse BECs and hepatocytes with chenodeoxycholic acid, biliatresone, acetaminophen (APAP) and free fatty acids (FFAs), thereby modelling cholestatic, toxic and metabolic injury patterns. Cells from healthy WT mice or from *Mdr2*^–/−^ mice with ongoing ductular reaction were used.^3^ Intriguingly, and in the absence of any stimuli, hepatocytes expressed higher *Orm2* than BECs in WT mice, while the *Orm2* expression tended to be downregulated in *Mdr2*^−/−^ hepatocytes but upregulated in *Mdr2*^−/−^ BECs (vs WT counterparts) (figure 1G and 3A). Gene (figure 1G and 3B) and protein (figure 1H1) expressions of ORM2 as well as cell damage levels (online supplemental figure S3C,D) were significantly upregulated in biliatresone-insulted BECs. In contrast, gene (figure 1G and 3E) and protein expressions (figure 1J and K) of ORM2 were significantly downregulated in APAP, FFA and biliatresone-exposed hepatocytes. Taken together, our data demonstrated that *Orm2* was upregulated in injured BECs but downregulated in injured hepatocytes, particularly during severe liver injury, which was associated with MoMF accumulation near ductular cells. We thus hypothesised ORM2 may represent a signal for the recruitment and/or activation of MoMFs in the biliary niche.

### A biliary niche-on-a-chip model demonstrates the potentiating roles of BEC-derived ORM2 in inflammation and hints towards more fibrogenesis

**Figure 2:**
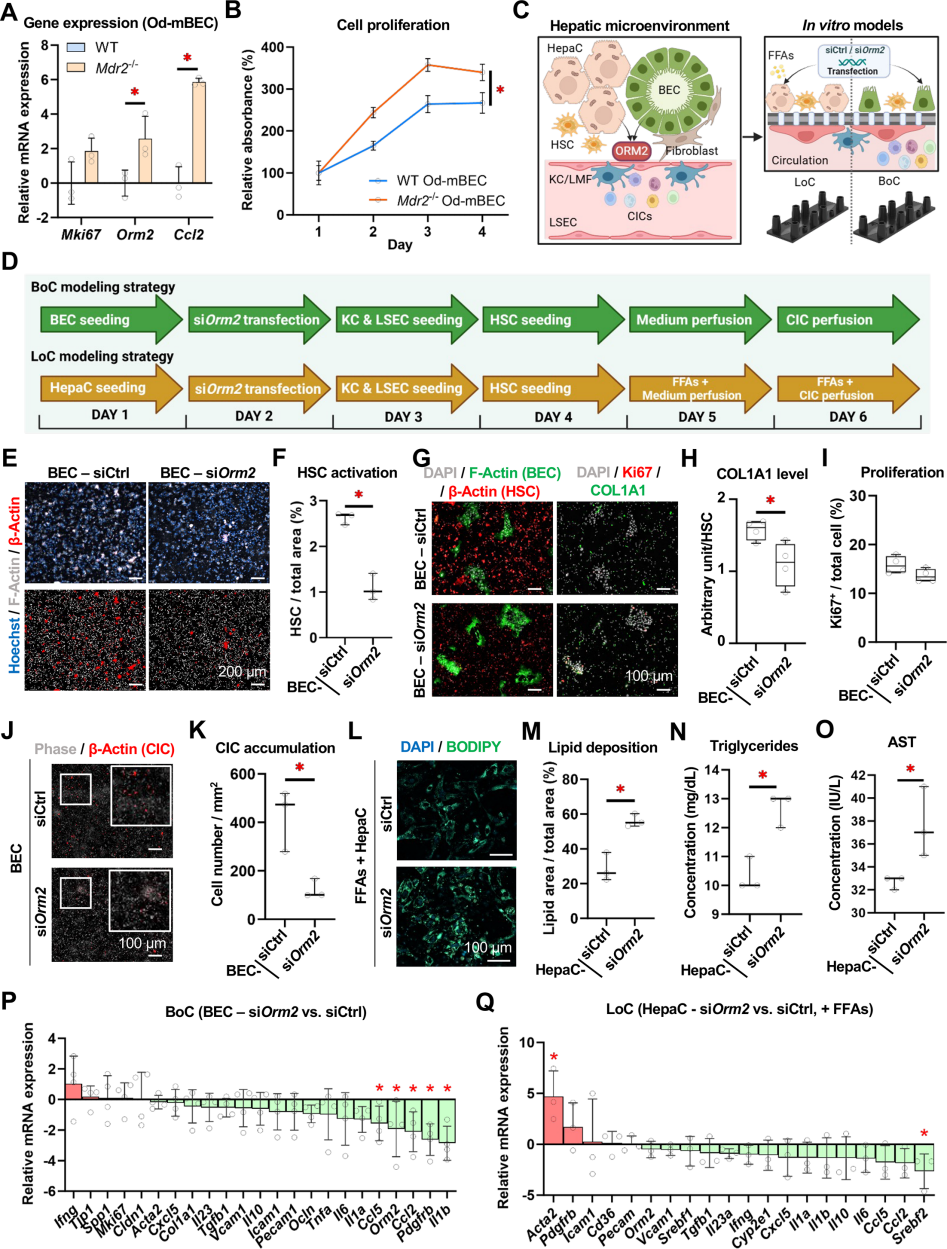
Evaluating influences of ORM2 in hepatic microenvironment in BoC and LoC models. (**A**) Gene expression of *Mki67*, *Orm2* and *Ccl2* in Od-mBECs generated from WT and *Mdr2*^−/−^ mice. (**B**) 4-day kinetics of cell proliferation in Od-mBECs generated from WT and *Mdr2*^−/−^ mice. (**C**) Scheme: using BoC and LoC models to recapitulate hepatic microenvironment and investigate influences of *Orm2*. (**D**) Experimental strategies of BoC and LoC were illustrated in a schematic figure. (**E**) HSC accumulation on siCtrl and si*Orm2* transfection in BECs was illustrated in fluorescent staining (F-actin^+^) and (**F**) quantitative analysis. (**G**) Collagen production (COL1A1^+^) and cell proliferation (Ki67^+^) were illustrated in fluorescent staining and (**H**) (**I**) quantitative analyses. (**J**) CIC accumulation (F-actin^+^) to the BoC membrane was illustrated in fluorescent staining and (**K**) quantitative analysis. (**L**) Lipid deposition (BODIPY^+^) was illustrated in fluorescent staining and (**M**) quantitative analysis. Levels of (**N**) triglycerides and (**O**) AST were measured in LoC cells on FFAs treatment (HepaC-siCtrl vs HepaC-si*Orm2*). (**P**) Gene expression of representative inflammation-associated, fibrogenesis-associated, adhesion-associated and tight junction-associated factors were measured in BoC cells (BEC-siCtrl vs BEC-si*Orm2*). (**Q**) Gene expression of representative inflammation-associated, fibrogenesis-associated, adhesion-associated factors were measured in LoC cells on FFAs treatment (HepaC-siCtrl vs HepaC-si*Orm2*). Sample sizes: n=3 per group. AST, aspartate transaminase; BoC, biliary niche-on-a-chip; CIC, circulating immune cell; COL1A1, collagen type I alpha 1 chain; Ctrl, control; FFA, free fatty acid; HFD, high-fat diet; HSC, hepatic stellate cell; LoC, liver-on-a-chip; (m)BEC, (mouse) biliary epithelial cell; (m)HepaC, (m)hepatocyte; Od, organoid-derived; ORM2, orosomucoid 2; WT, wild type. Unpaired Student’s t-tests were performed. *p<0.05 as indicated or as compared with controls.

To investigate BEC biology in the context of ductular reaction, WT and *Mdr2*^−/−^ mouse organoid-derived (mOd)-BECs were generated and characterised. *Mdr2*^−/−^ Od-mBECs had higher levels of *Mki67*, *Orm2* and *Ccl2* gene expression (figure 2A), together with higher proliferation rates, which are reflective of their reactive phenotype (vs WT) being preserved for several passages after their isolation from the liver (figure 2B). Thus, *Mdr2*^−/−^ Od-mBECs were used in subsequent experiments as a cellular model of injury-related higher *Orm2* expression. Transfection of *Mdr2*^−/−^ Od-mBECs with si*Orm2* suppressed gene and protein expression of *Orm2* but not *Orm1* (online supplemental figure S4A–C). Tailored multicellular in vitro biochips were designed to mimic the sinusoidal and the biliary immunological niches (LoC^23^ and BoC, respectively) (figure 2C–D, S4D). *Orm2* silencing in Od-mBECs also seemed to result in reduced hepatic stellate cell (HSC) activation, as reflected by reduced area covered by pre-labelled F-actin^+^ HSCs (figure 2E–F) and collagen expression (COL1A1^+^) in the BoC (figure 2G–H), whereas no significant differences were observed in cell proliferation (Ki67^+^) (figure 2I). Furthermore, pre-labelled circulating immune cells (CIC) were perfused in the biochips, and their accumulation in the BoC was lower on *Orm2* silencing in Od-mBECs as compared with scramble siRNA (figure 2J–K). Similarly, si*Orm2* transfection effectively suppressed gene and protein expression of *Orm2* but not *Orm1* in mouse primary hepatocytes (online supplemental figure S4E–G). On *Orm2* silencing, FFA treatment induced higher lipid accumulation in mouse hepatocytes in the LoC (figure 2L–M). In line with this, levels of triglycerides (figure 2N) and aspartate transferase (figure 2O) were significantly increased in *Orm2*-silenced hepatocytes exposed to FFAs. As previously reported, alanine aminotransferase (ALT) levels did not reach detection thresholds in the LoC.^23^

Moreover, BoC gene expression analyses indicated the significant downregulation of *Ccl5*, *Orm2*, *Ccl2*, *Pdgfrb* and *Il1b* on *Orm2* suppression in Od-mBECs (figure 2P). In the LoC, we denoted a significant upregulation of *Acta2*, suggesting fibrogenic HSC activation and downregulation of *Srebf2*, a sign of decreased lipid metabolism during *Orm2* silencing in hepatocytes (figure 2Q). Taken together, our data indicate that hepatocyte-derived ORM2 limits cellular injury and lipid accumulation in hepatocytes under stress conditions, while reactive *Mdr2*^–/–^ BEC-derived ORM2 promotes inflammation and potentially fibrogenesis in the biliary niche.

**Figure 3:**
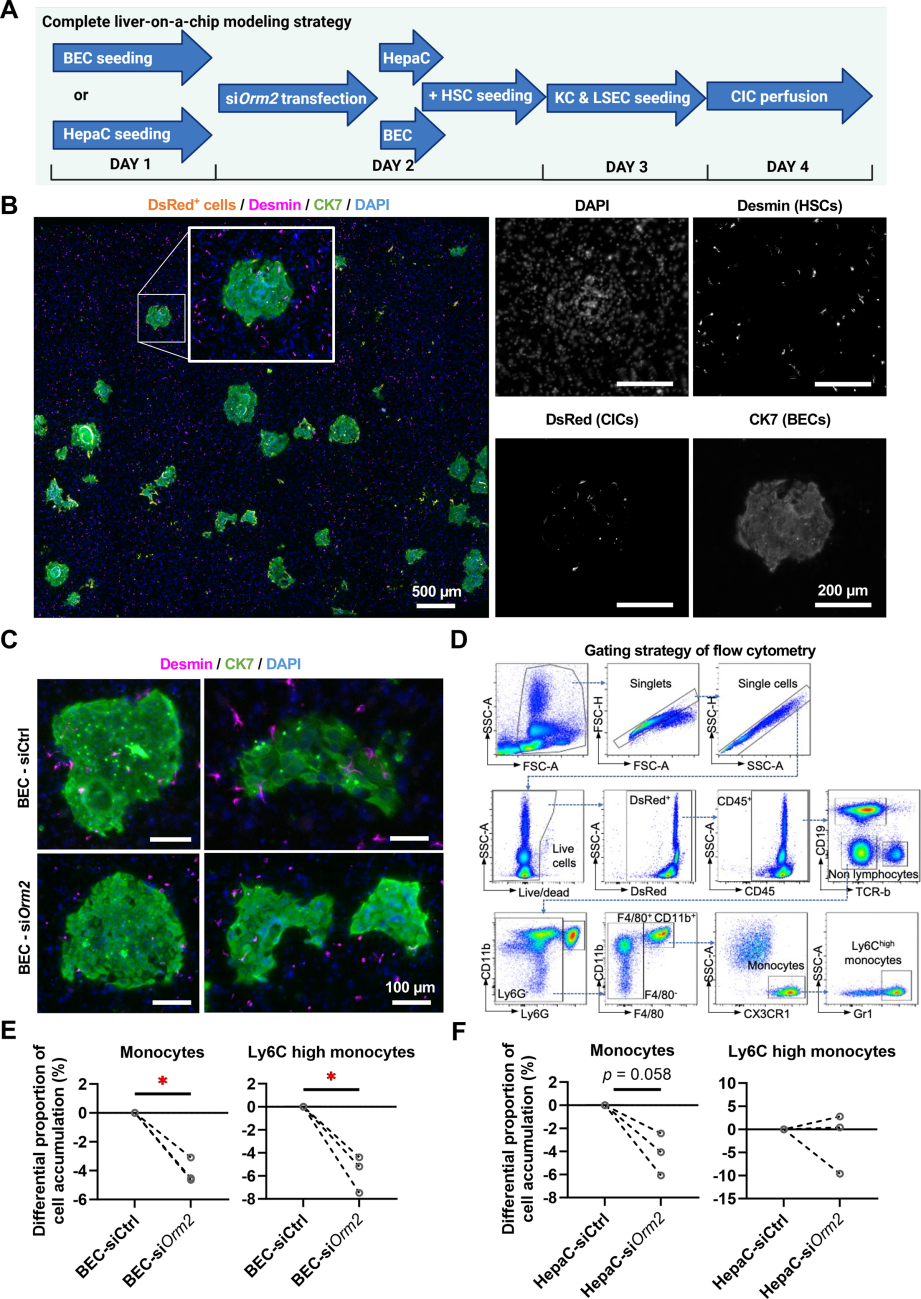
A complete liver-on-a-chip containing both BECs and hepatocytes suggests that ORM2 regulates pathogenic monocyte recruitment. (**A**) Seeding sequence and experimental approach to generate a complete liver-on-a-chip. (**B**) Cell seeding and identities were confirmed by immunocytochemistry in the complete liver-on-a-chip. (**C**) Enlarged areas depict desmin^+^ and CK7^+^ cell. (**D**) Gating strategy applied for flow cytometry evaluation of circulating immune cells in the complete liver-on-a-chip. Monocytes (live CD45^+^CD19^−^TCR-beta^−^Ly6G^−^F4/80^+^CD11b^+^CX3CR1^high^) and Ly6C^high^ monocytes were numbered in the complete liver-on-a-chip after either (**E**) BEC-targeted, or (**F**) hepatocyte-targeted *Orm2* silencing. BECs, biliary epithelial cells; CICs, circulating immune cells; CK7, cytokeratin 7; HSCs, hepatic stellate cells; ORM2, orosomucoid 2. Paired Student’s t-tests were used. *p<0.05 as indicated.

To better dissect the relevance of hepatocyte-derived versus BEC-derived ORM2, we next constructed a complete LoC model that contains both primary hepatocytes and *Mdr2*^–/–^ reactive BECs (expressing high levels of *Orm2*) (figure 3A). We first confirmed that all cells seeded well and organised properly in the biochip. As shown in figure 3B, we applied a gentle digestion to BEC organoids before seeding into the biochip, which resulted in BECs forming agglomerates and thus, resembling portal areas observed in vivo. Next, we silenced *Orm2* either in hepatocytes or in BECs. We noticed that desmin^+^ HSCs tended to accumulate in the surroundings of BECs, particularly when *Orm2* was expressed (figure 3C). Besides, we observed a preferential accumulation of CICs in the BEC areas (figure 3B), and thus aimed at characterising the nature of those CICs by flow cytometry. Considering that si*Orm2* significantly reduced *Ccl2* expression in the BoC, we designed a flow cytometry gating strategy to assess monocyte recruitment and more specifically, that of Ly6C^high^ monocytes, which express the CCL2 receptor CCR2 and are classically associated with pathogenic processes in liver diseases.^41^ Interestingly, our data showed that BEC-targeted but not hepatocyte-targeted *Orm2* silencing led to a reduction in Ly6C^high^ monocyte mobilisation to the biochip (figure 3E and F). This observation further suggested that macrophages may represent a privileged interaction partner of BECs during liver disease.

### ORM2 reshapes transcriptome profiles and biological functions of liver macrophages

**Figure 4:**
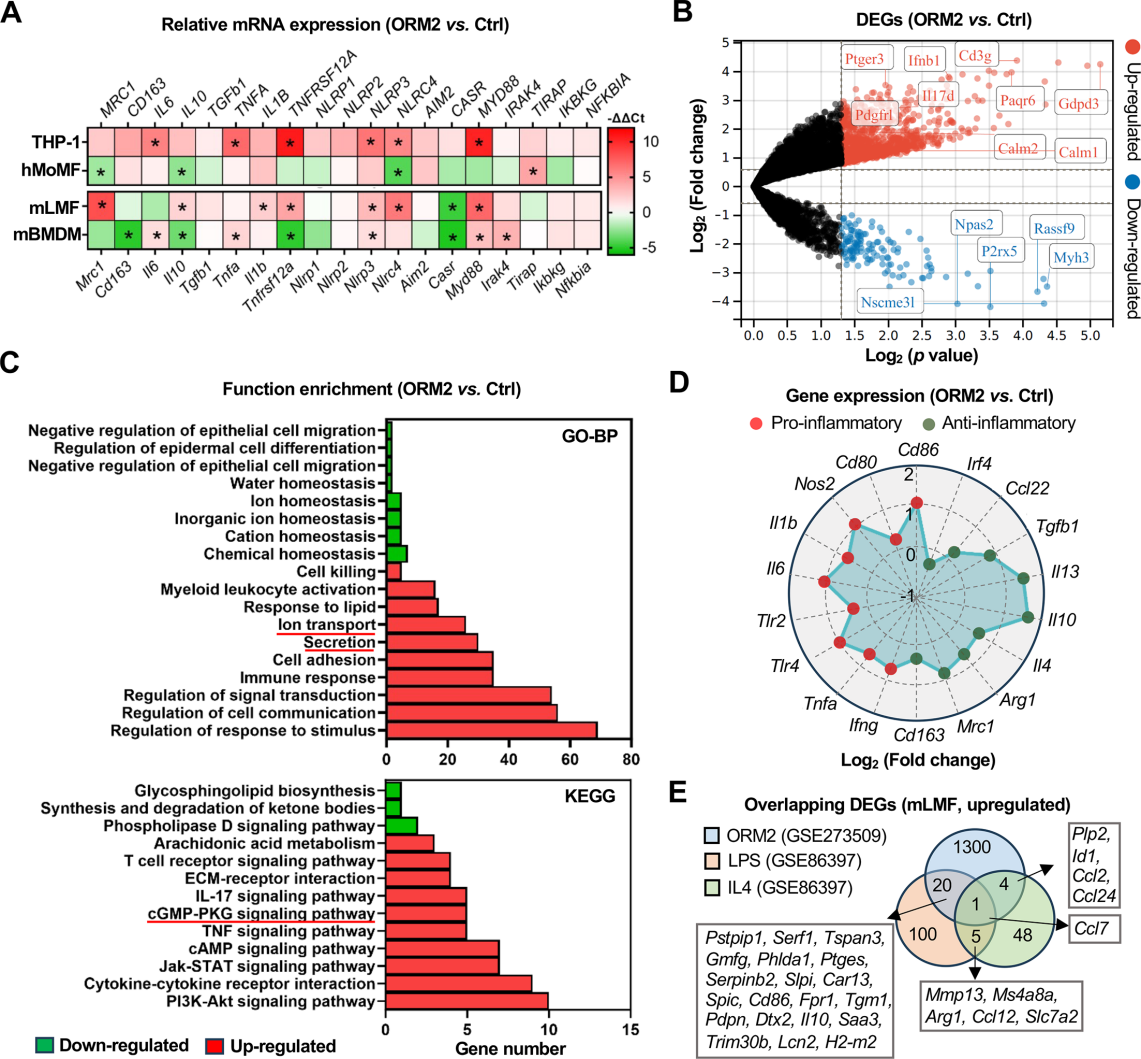
ORM2 reshapes transcriptome profiles in liver macrophages. (**A**) Gene expression of macrophage phenotype-associated markers was measured in human THP-1 cell line, hMoMF, mLMF and mBMDM (ORM2-treated vs Ctrl). (**B**) Significantly upregulated and downregulated DEGs in mLMF by bulk RNA-seq analysis were illustrated in the volcano plot (ORM2-treated vs Ctrl). (**C**) Function enrichment analysis (GO-BP and KEGG) on significantly upregulated and downregulated DEGs in mLMF by bulk RNA-seq analysis was illustrated (ORM2-treated vs Ctrl). (**D**) Gene expression changes of pro-inflammatory and anti-inflammatory macrophage phenotype-associated markers in mLMF by bulk RNA-seq analysis were illustrated in the radar plot (ORM2-treated vs Ctrl). (**E**) Overlapped significantly upregulated DEGs in mLMF from diverse bulk RNA-seq data sets (GSE273509 and GSE86397) were illustrated (vs Ctrl). Sample sizes: (**A**) n=4 per group; (**B–E**) ORM2-treated (n=4) versus Ctrl (n=5). DEG, differentially expressed gene; GO-BP, Gene Ontology-biological processes; hMoMF, human monocyte-derived macrophages; KEGG, Kyoto Encyclopedia of Genes and Genomes; mLMF, mouse liver macrophages; mBMDM, mouse bone marrow-derived macrophages; mRNA, messenger RNA; ORM2, orosomucoid 2; RNA-seq, RNA sequencing. Unpaired Student’s t-tests were used. *Represents p<0.05.

**Figure 5:**
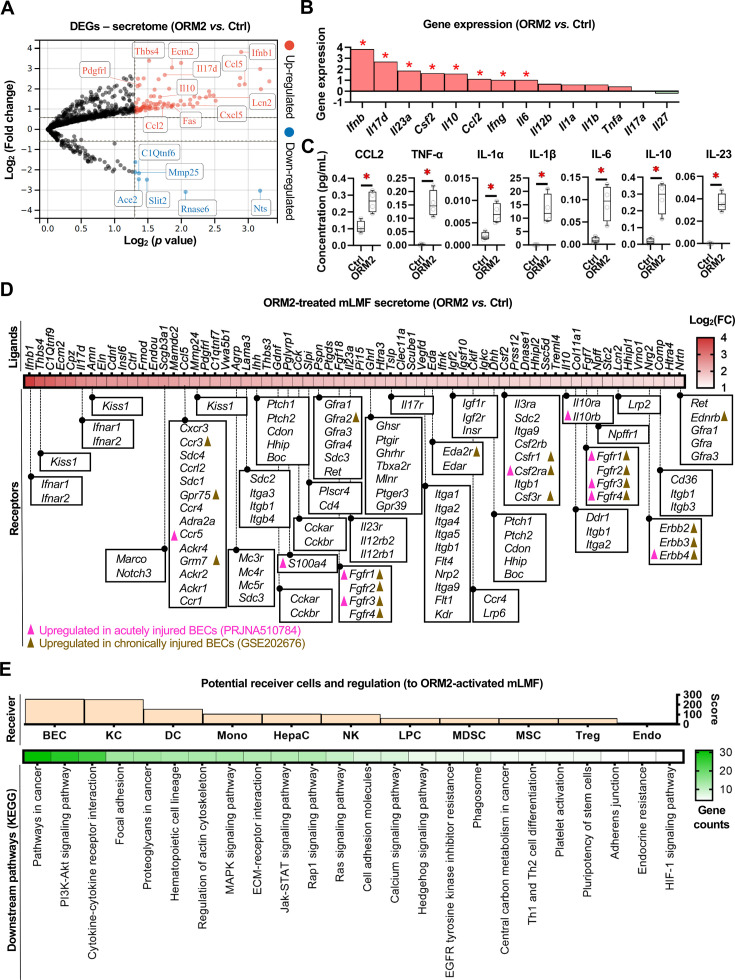
ORM2 reshapes secretome profiles in liver macrophages. (**A**) Significantly upregulated and downregulated DEGs (encoding secreted proteins) in mLMF by bulk RNA-seq analysis were illustrated in the volcano plot (ORM2-treated vs Ctrl). (**B**) Gene expression and (**C**) cytokine concentration of several inflammatory cytokines in mLMF were illustrated (ORM2-treated vs Ctrl). (**D**) Ligand-receptor prediction from ORM2-activated mLMF were depicted in a flow chart. (**E**) Function and cell type enrichments were depicted in a flow chart. Sample sizes: (**A and B**) ORM2-treated (n=4) versus Ctrl (n=5); (**C**) n=4 per group. DEGs, differentially expressed genes; IL, interleukin; KEGG, Kyoto Encyclopedia of Genes and Genomes; mLMF, mouse liver macrophages; ORM2, orosomucoid 2; RNA-seq, RNA sequencing; TNF, tumour necrosis factor. Unpaired Student’s t-tests were used. *Represents p<0.05.

Since our results point towards spatially-restricted and disease-associated BEC and macrophage interactions through ORM2, we next investigated the direct effects of ORM2 on macrophages of different origins. Primary human blood monocyte-derived macrophages (hMoMF), mouse liver macrophages (mLMF) and mouse bone marrow-derived macrophages (mBMDM), together with the (differentiated) human monocyte cell line (THP-1) were obtained for gene expression profiling. In response to ORM2, mLMF and THP-1 cells showed increased gene expression of markers classically associated with pro-inflammatory and anti-inflammatory polarisation profiles (including *Mrc1*, *Il10*, *Tnfrsf12a*, *Nlrp3*, *Nlrc4* and *Myd88*), as opposed to hMoMF and mBMDM that mostly showed reduced gene expressions (figure 4A and 5). Based on this exploratory screening that indicated a stronger response of mLMF, we next performed bulk transcriptome sequencing on mLMF exposed to ORM2. The top 20 upregulated and downregulated DEGs were identified and confirmed profound changes in the mLMF transcriptome (figure 4B, online supplemental figure S6). Functional enrichment analyses (Gene Ontology-biological processes and Kyoto Encyclopedia of Genes and Genomes (KEGG)) indicated the activation of various cellular functions (eg, ion transport, secretion, immune response) and signalling pathways (eg, cGMP-PKG, IL-17, TNF, cAMP, Jak-STAT and PI3K-Akt pathways) in response to ORM2 treatment (figure 4C). Moreover, we confirmed that ORM2 induced the upregulation of both pro-inflammatory and anti-inflammatory macrophage markers (figure 4D). Interestingly, few DEGs were overlapping with those from either lipopolysaccharides (LPS)-stimulated or IL-4-stimulated mLMF,^42^ hence suggesting a unique ORM2-induced mLMF activation profile (figure 4E).

To further decipher cellular crosstalk, we next investigated the changes in the secretome-associated DEGs of mLMF exposed to ORM2 (figure 5A). Gene expression and/or protein secretion of CCL2, tumour necrosis factor (TNF)-α, interleukin (IL)-1α, IL-1β, IL-6, IL-10 and IL-23 were significantly enhanced by ORM2 in mLMF (figure 5B,C, online supplemental figure S7). In addition, CellTalkDB^43^ was used to predict potential receptor-ligand interactions. First, receptors of which gene expression was upregulated in acutely or chronically injured BECs^8 27^ were identified and aligned with their ligands found in ORM2-treated mLMF (figure 5D). This approach highlighted a number of top matches between injured BECs and ORM2-treated mLMF such as *Ccr5-Ccl5* and *Fgf-receptors*, pathways involved in fibrogenesis, metabolic disorders and even tumourigenesis.^44 45^ Next, CellMarker^46^ and Enrichr^47^ databases were employed to predict ORM2-activated mLMF secretome responder cells based on gene enrichment scores, and then the KEGG database was used to highlight the predicted dominant downstream pathways (figure 5E). In a nutshell, data suggests that ORM2-activated mLMFs have broad effects on multiple cell types within their hepatic microenvironment yet, BECs and Kupffer cells (KCs) stand as the main ligand-responsive cells, with effects predicted to be related mostly to cell cycle and cellular crosstalk, respectively, further highlighting positive feedback loops sustaining ductular reaction, that is, BEC proliferation and macrophage activation.

**Figure 6:**
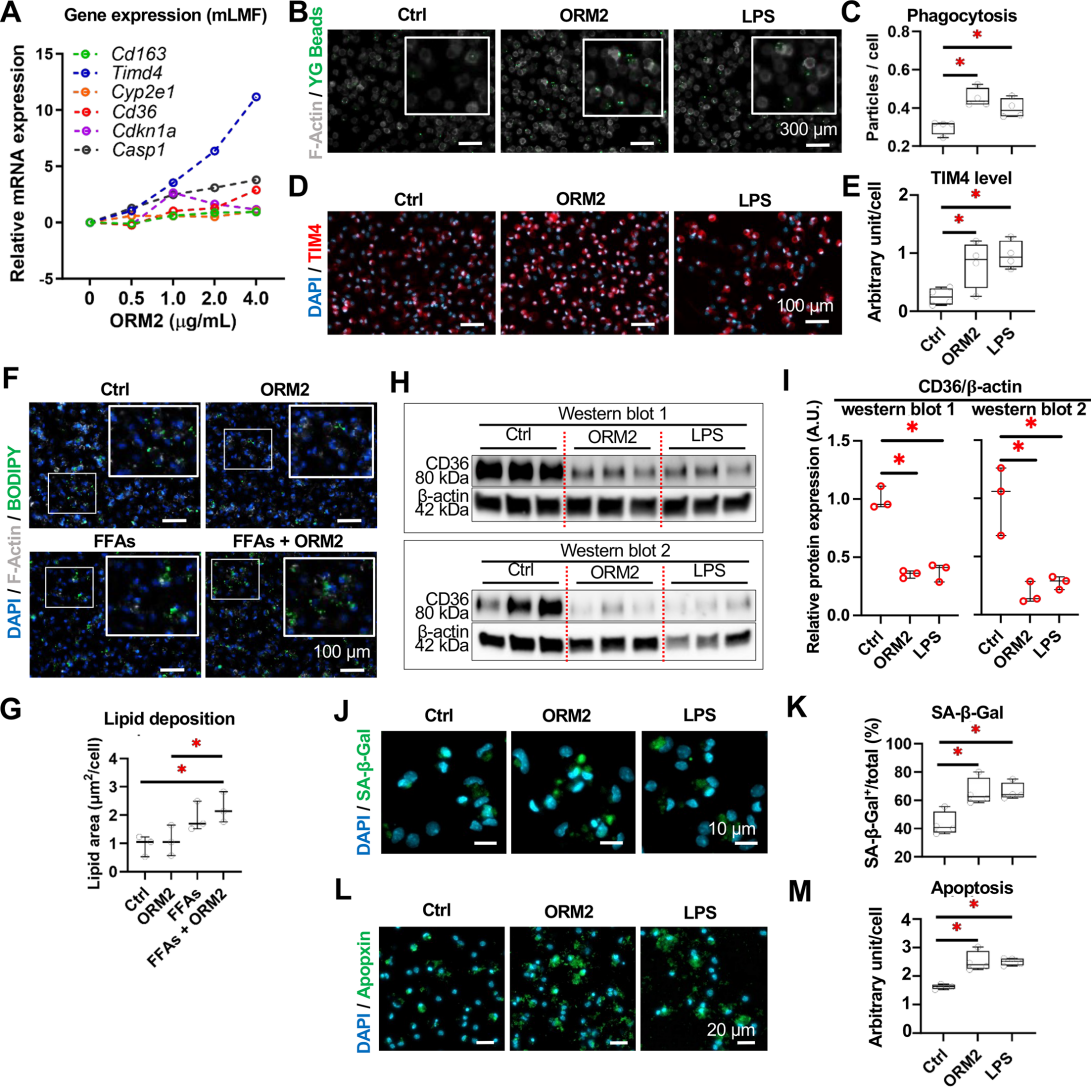
ORM2 promotes phagocytosis, lipid deposition and cell stress in liver macrophages. (**A**) Gene expression of *Cd163*, *Timd4*, *Cyp2e1*, *Cd36*, *Cdkn1a* and *Casp1* in mLMF on ORM2 treatments (0, 0.5, 1.0, 2.0 and 4.0 µg/mL) was measured (ORM2-treated vs Ctrl). (**B**) Phagocytosis/bead capture (YG^+^) of mLMF on Ctrl, ORM2 and LPS treatments was illustrated in fluorescent staining and (**C**) quantitative analysis. (**D**) Protein expression of TIM4 in mLMF on Ctrl, ORM2 and LPS treatments was illustrated in fluorescent staining and (**E**) quantitative analysis. (**F**) Lipid deposition of mLMF on Ctrl, ORM2, FFAs and FFAs+ORM2 treatments was illustrated in fluorescent staining and (**G**) quantitative analysis. (**H**) Protein expression of CD36 in mLMF on Ctrl, ORM2 and LPS treatments was assessed by western blot in two independent experiments (both displayed) and (**I**) quantitative analysis was performed. (**J**) DNA damage (Sa-β-Gal^+^) of mLMF on Ctrl, ORM2 and LPS treatments was illustrated in fluorescent staining and (**K**) quantitative analysis. (**L**) Cell apoptosis (Apopxin^+^) of mLMF on Ctrl, ORM2 and LPS treatments was illustrated in fluorescent staining and (**M**) quantitative analysis. Sample sizes: n=3–4 per group. CD36, cluster of differentiation 36; FFAs, free fatty acids; ITPR2, inositol 1,4,5-trisphosphate receptor type 2; LPS, lipopolysaccharides; mLMF, mouse liver macrophages; ORM2, orosomucoid 2; SA-β-Gal, senescence-associated beta-galactosidase; TIM4, T-cell/transmembrane immunoglobulin and mucin domain containing 4; YG, yellow green fluorescence. One-way analysis of variance followed by Tukey’s multiple comparison tests were performed. *p<0.05 as indicated or as compared with controls.

We next aimed at further elucidating ORM2 effects on macrophage biological functions. We first evidenced a dose-response effect of ORM2 treatment on mLMF *Timd4* expression (figure 6A). This was further evidenced by increased phagocytosis capacity (figure 6B–C) and protein levels of T-cell/transmembrane immunoglobulin and mucin domain containing 4 (TIM4) (figure 6D–E) on ORM2 treatment, similarly to what is observed after LPS exposure. Liver macrophages participate in lipid metabolism via CD36,^48^ and interestingly, we noted that lipid storage in macrophages was not increased by ORM2 treatment (FFAs+ORM2 vs FFAs) (figure 6F–G). However, CD36 protein levels were significantly decreased on ORM2 and LPS treatments in mLMF (figure 6H–I, raw western blot data in online supplemental figure S8). In addition, ORM2 increased senescence-associated beta-galactosidase (SA-β-GAL^+^) and apoptosis (Apopxin^+^) in mLMF similarly to LPS (figure 6J–M). Multispectral flow cytometry was used to further characterise macrophage phenotypes of mLMF, mBMDM and hMoMF (online supplemental figure S9A,B). Although conventional marker expressions were unchanged on mLMF exposed to ORM2 and LPS treatments (online supplemental figure S9C), mBMDM and hMoMF tended to express protein markers associated with pro-inflammatory phenotypes (online supplemental figure S9D,E). Taken together, ORM2 reshapes transcriptome profiles and alters the cellular phenotype (including promoting secretome, phagocytosis, lipid handling and cell stress) in mLMFs, although those changes may not be reflected by conventional extracellular protein markers.

### ORM2 reprogrammes liver macrophage functions through ITPR2/CALM-dependent calcium pathway

**Figure 7:**
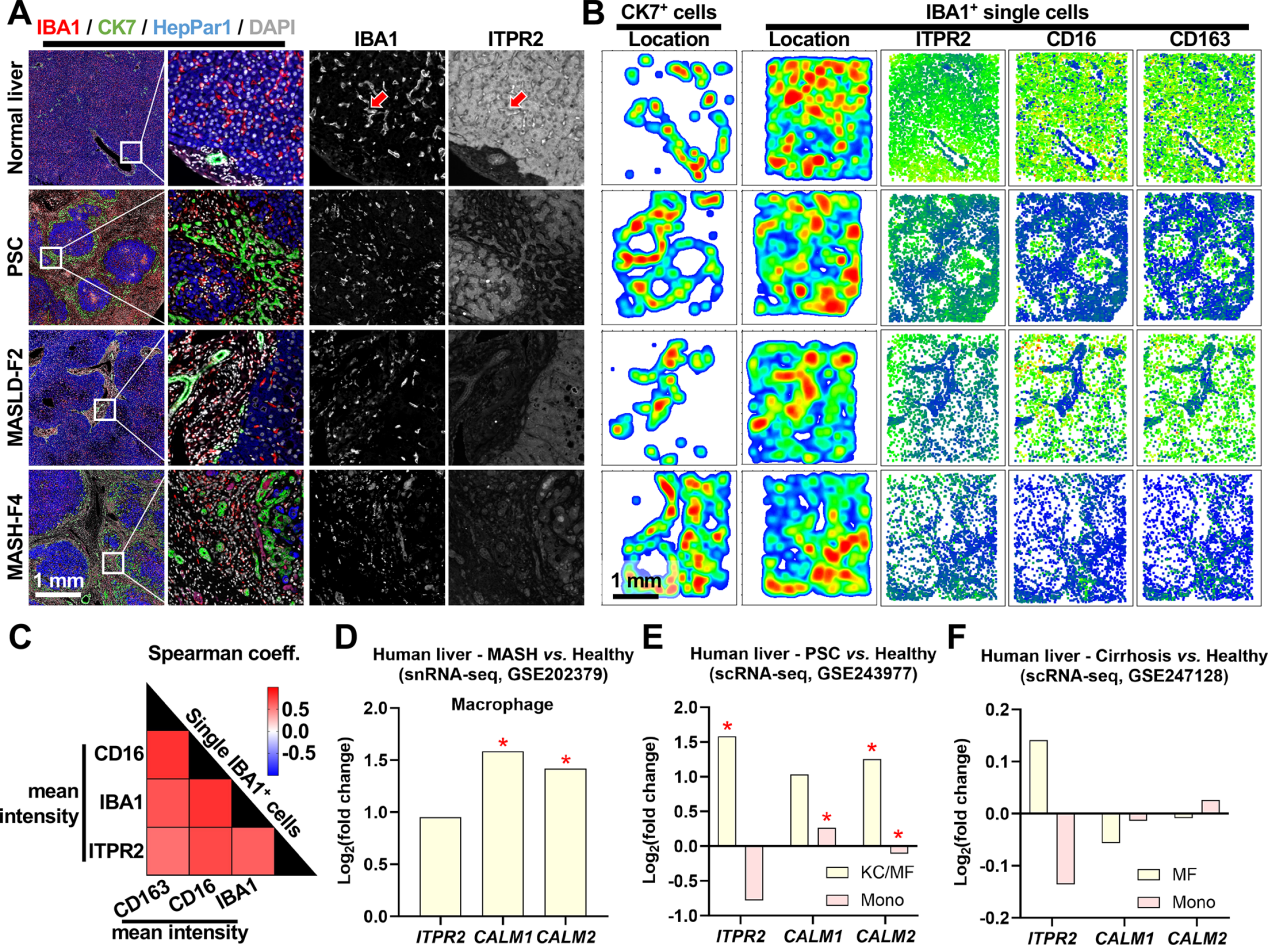
Multiplex immunofluorescence and single-cell transcriptomics reveal heterogeneous ITPR2 expression in liver macrophages during liver diseases. (**A**) Multiplex immunofluorescence was applied to identify hepatocytes (HepPar1^+^), cholangiocytes (CK7^+^) and macrophages (IBA1^+^), together with ITPR2 expression in human normal, PSC, MASLD-F2 and MASH-F4 liver samples. Red arrows point toward ITPR2-expressing macrophages. (**B**) Large scan multiplex immunofluorescence images shown in (**A**) were used for CK7^+^ and IBA1^+^ cell distribution analysis (heatmaps), and singular IBA1^+^ cell relative staining intensities for ITPR2, CD16 and CD163 (dot plots, green: high staining intensity, blue: low staining intensity). (**C**) Spearman’s correlation was used to evaluate the correlation between single cell immunostaining intensities of the indicated proteins in IBA1^+^ cells present in the images shown in panels A and B. All markers showed significant correlations. Differential gene expressions were investigated in the indicated data sets for ITPR2, CALM1 and CALM2 in monocyte and macrophage populations in human (**D**) MASH, (**E**) PSC and (**F**) liver cirrhosis (all vs healthy). F2/4, fibrosis stage 2/4; KC, Kupffer cells; ITPR2, inositol 1,4,5-trisphosphate receptor type 2; MASH, metabolic dysfunction-associated steatohepatitis; MASLD, metabolic dysfunction-associated steatotic liver disease; MF, macrophages; Mono: monocytes; PSC, primary sclerosing cholangitis; sc/snRNA-seq, single cell/single nuclei RNA sequencing. (**D**–**F**) Unpaired Student’s t-tests were used. *Represents p<0.05.

**Figure 8:**
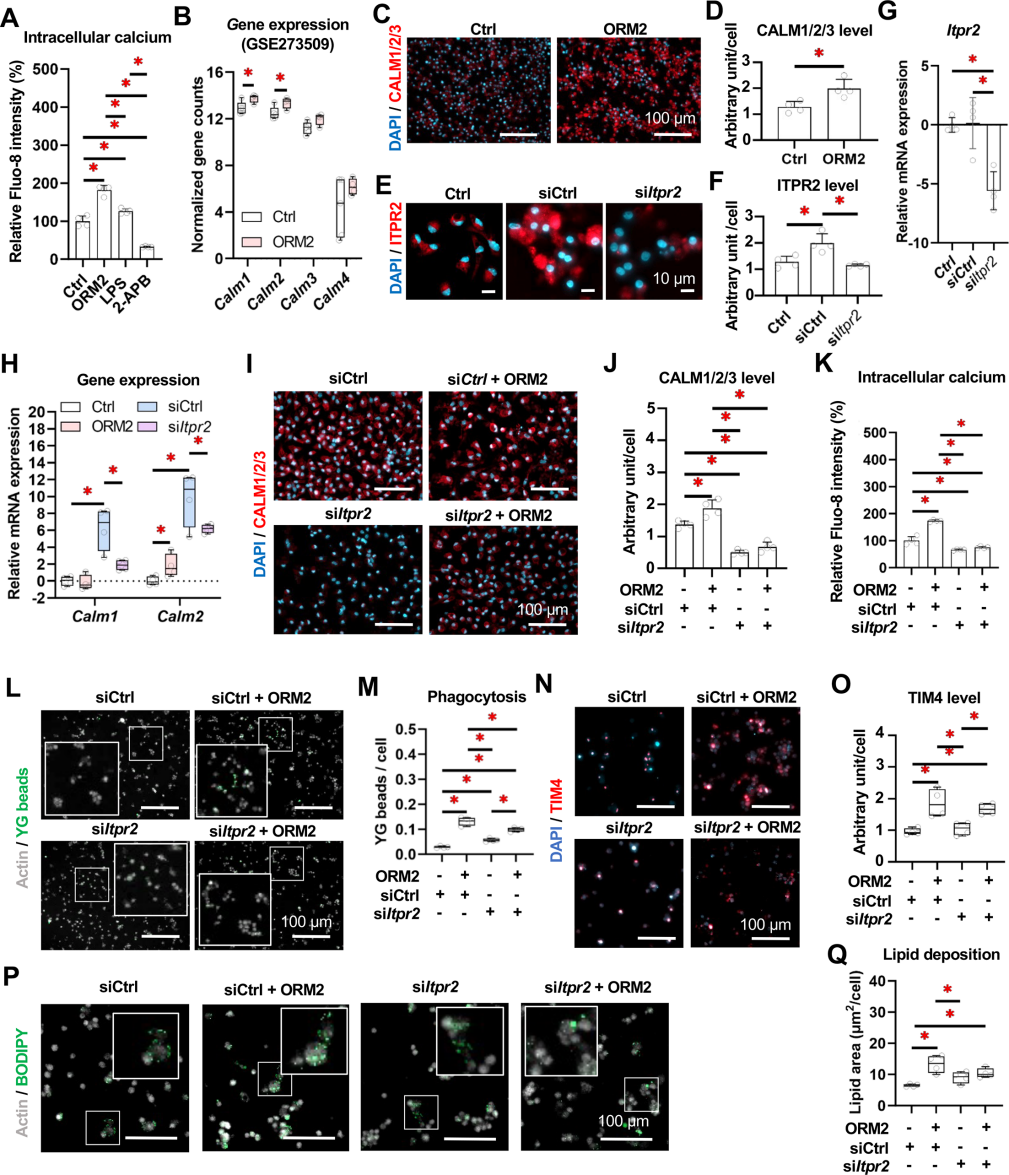
ORM2 alters macrophage functions via ITPR2/CALMs-dependent calcium pathway. (**A**) Intracellular calcium levels of mLMF on Ctrl, ORM2, LPS and 2-APB treatments were measured. (**B**) Gene expression of *Calm1/2/3/4* in mLMF was illustrated from bulk RNA sequencing analysis (ORM2-treated vs Ctrl). (**C**) Protein expression of CALM1/2/3 in mLMF was illustrated in fluorescent staining and (**D**) quantitative analyses (ORM2-treated vs Ctrl). (**E**) Protein expression of ITPR2 in mLMF on Ctrl, siCtrl and si*Itpr2* transfection was illustrated in fluorescent staining and (**F**) quantitative analyses. (**G**) Gene expression of *Itpr2* in mLMF on Ctrl, siCtrl and si*Itpr2* transfection was measured. (**H**) Gene expression of *Calm1* and *Calm2* in mLMF on Ctrl, siCtrl and si*Itpr2* transfection was measured. (**I**) Protein expression of CALM1/2/3/4 in mLMF on Ctrl, siCtrl and si*Itpr2* transfection was illustrated in fluorescent staining and (**J**) quantitative analyses. (**K**) Intracellular calcium levels of mLMF on Ctrl, siCtrl and si*Itpr2* transfection were measured. (**L**) Phagocytosis/bead capture (YG^+^) of mLMF on siCtrl, siCtrl+ORM2, si*Itpr2* and si*Itpr2*+ORM2 was illustrated in fluorescent staining and (**M**) quantitative analysis. (**N**) Protein expression of TIM4 in mLMF on siCtrl, siCtrl+ORM2, si*Itpr2* and si*Itpr2*+ORM2 was illustrated in fluorescent staining and (**O**) quantitative analysis. (**P**) Lipid deposition of mLMF on siCtrl, siCtrl+ORM2, si*Itpr2* and si*Itpr2*+ORM2 was illustrated in fluorescent staining and (**Q**) quantitative analysis. Sample sizes: n=3–4 per group. 2-APB, 2-aminoethoxydiphenyl borate; CALM, calmodulin; ITPR2, inositol 1,4,5-trisphosphate receptor 2; LPS, lipopolysaccharides; mLMF, mouse liver macrophages; ORM2, orosomucoid 2; TIM4, T-cell/transmembrane immunoglobulin and mucin domain containing 4; YG, yellow green fluorescence. One-way analysis of variance followed by Tukey’s multiple comparison tests were performed. *p<0.05 as indicated or as compared with controls.

Considering the broad effects of ORM2 on macrophage transcriptome and secretome, we aimed at identifying the underlying intracellular signalling pathway in macrophages, which may represent an attractive target for pharmacological intervention. It had been recently demonstrated that ORM2 binds to inositol 1,4,5-trisphosphate receptor type 2 (ITPR2) to activate calcium signalling pathways and enhance metabolism capacities in hepatocytes.^20^ Multiplex immunofluorescence showed that ITPR2 is broadly expressed in hepatocytes, cholangiocytes and macrophages in normal liver (figure 7A). Single cell-resolved image analyses revealed that liver macrophages showing markers of functional scavenging abilities, that is, potent expression of CD16 and CD163, were also expressing higher levels of ITPR2 (figure 7B and C). This data also shows that in normal liver, ITPR2 expression is mostly concentrated on liver macrophages located in parenchymal areas (assumed to be KCs), while during disease ITPR2 expression expands towards the portal areas, potentially reflecting freshly recruited macrophage activation. Furthermore, single-cell/single-nuclei data sets from human MASH, PSC and cirrhotic livers revealed that expressions of *ITPR2*, *CALM1* and *CALM2* were upregulated in macrophage populations (MASH or PSC vs healthy), but not in monocyte populations (figure 7D and E). However, no significant changes in expression of *ITPR2*, *CALM1* and *CALM2* were found in cirrhotic livers (vs healthy) (figure 7F). Intriguingly, we found that ORM2 and LPS (used as a positive control) significantly enhance cytosol calcium levels in mLMFs isolated from WT mouse livers (figure 8A). Furthermore, gene expressions and protein levels of CALM1 and 2 were upregulated on ORM2 treatment in mLMF (vs Ctrl) (figure 8B,D). To demonstrate the involvement of ITPR2 in mLMF calcium-dependent signalling pathways, we first validated that an siRNA-mediated knock-down of *Itpr2* effectively suppressed protein levels and gene expression of ITPR2 in mLMFs (figure 8E–G). Notably, siCtrl also increased ITPR2 expression, a phenomenon likely due to the effects of Lipofectamine on intracellular processes.^49^ Next, we evidenced that gene expression and protein levels of CALM1 and 2 were suppressed on *Itpr2* silencing in mLMF (vs siCtrl) (figure 8H–J). Besides, *Itpr2* silencing suppressed cytosol calcium transport in mLMF (vs siCtrl) (figure 8K). Importantly, ORM2-enhanced phagocytosis (YG beads^+^ and TIM4^+^) and lipid intake (BODIPY^+^) were compromised in mLMFs after *Itpr2* silencing (figure 8L–Q). In line, ORM2-induced mLMF SA-β-GAL signal was not affected, and apoptosis (Apopxin^+^) was reduced on *Itpr2* silencing (online supplemental figure S10A–D). The gene expression of *Itpr2*, *Timd4*, *Cd36* and *Tgfb1* was suppressed in mLMF while the gene expression of *Il10* was increased after *Itpr2* silencing. The expression of most of the assessed genes was not significantly altered by ORM2 treatment in combination with *Itpr2* silencing except *Il1b* and *Tgfb1* (online supplemental figure S10E). In addition, results from multicellular coculture experiments using Od-mBECs, LMFs and HSCs demonstrated that BEC-derived ORM2 enhanced CALM1/2/3 levels in LMFs and fibrogenesis potential in HSCs (online supplemental figure S11A–C). Taken together, cholangiocyte-derived ORM2 upregulates LMF calcium intake and reprogrammes LMF functions towards a distinctive phagocytic and pro-inflammatory phenotype through the ITPR2/CALM axis.

### ORM2-activated liver macrophages signal back to suppress biliary cell proliferation and may promote fibrogenesis

Since BEC-macrophage spatial co-localisation is a typical observation in the biliary niche, and to identify the implications of ORM2 on BEC and hepatocyte macrophage crosstalk, we silenced *Orm2* in mouse primary hepatocytes and in Od-mBECs to obtain BEC and hepatocyte conditioned media (CM) to which mLMFs and mBMDM were exposed. Gene expressions of *Cyp2e1* and *Mrc1* were upregulated while the gene expression of *Nos2* and *Il1b* were downregulated in mLMFs cultured with CM from mHepaC-si*Orm2* (vs mHepaC-Ctrl) (online supplemental figure S12A). Moreover, gene expression of *Mrc1* was upregulated while gene expression of *Cd36*, *Nos2* and *Il1b* were downregulated in mLMFs cultured with CM from Od-mBEC-si*Orm2* (vs Od-mBEC-Ctrl) (online supplemental figure S12B). In contrast, we observed few significant changes in gene expression in mBMDMs (online supplemental figure S12C,D). In line with the previous data, these results implied that ORM2 promotes a singular activation phenotype of mLMFs, despite some differences between BEC-derived and hepatocyte-derived CM likely due to unidentified co-secreted factors. This data suggests that mLMF are the main responders to BEC-derived ORM2 during ductular reaction.

**Figure 9:**
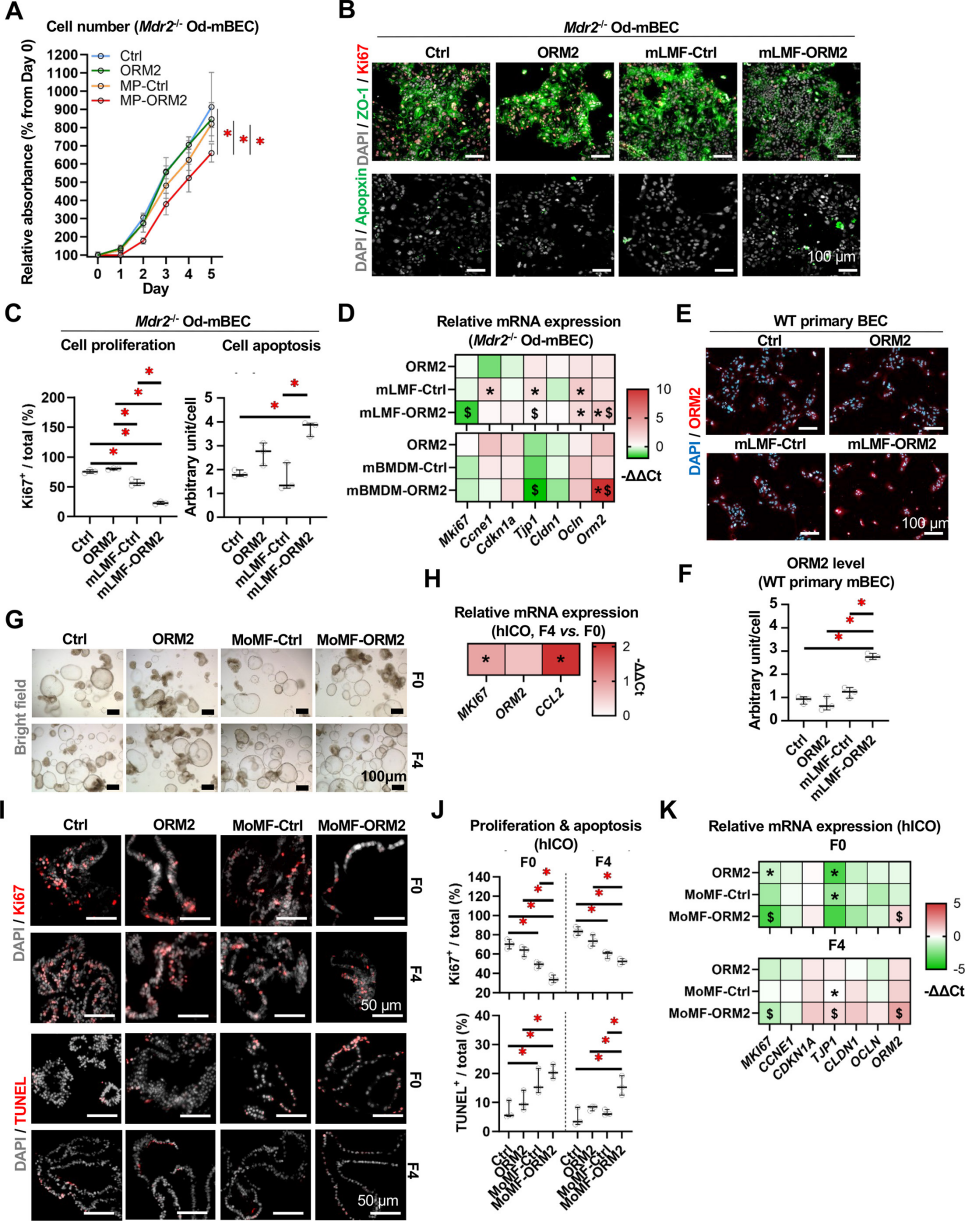
ORM2-activated liver macrophages suppress biliary cell repair. (**A**) 5-day kinetics of *Mdr2*^−/−^ Od-mBEC numbers on Ctrl, ORM2, CM from Ctrl mLMF and ORM2-treated mLMF were measured. (**B**) Cell proliferation (Ki67^+^) and apoptosis (Apopxin^+^) in *Mdr2*^−/−^ Od-mBECs on Ctrl, ORM2, CM from Ctrl mLMF and ORM2-treated mLMF was investigated by fluorescent staining and (**C**) quantitative analyses. (**D**) Gene expressions of *Mki67*, *Ccne1*, *Cdkn1a*, *Tjp1*, *Cldn1*, *Ocln* and *Orm2* in *Mdr2*^−/−^ Od-mBECs on Ctrl, ORM2, CM from Ctrl mLMF and ORM2-treated mLMF were measured. (**E**) Protein expression of ORM2 in mouse primary BECs on Ctrl, ORM2, CM from Ctrl mLMF and ORM2-treated mLMF was illustrated in fluorescent staining and (**F**) quantitative analyses. Patient (liver fibrosis stage 0 and 4)-derived hICOs (one line per disease condition) were obtained and cultivated. (**G**) Morphology of patient (liver fibrosis stage 0 and 4)-derived hICOs on Ctrl, ORM2, CM from Ctrl human MoMF and ORM2-treated human MoMF was illustrated. (**H**) Gene expressions of *MKI67*, *ORM2* and *CCL2* in patient (liver fibrosis stage 0 and 4)-derived hICOs were measured (F4 vs F0). (**I**) Cell proliferation (Ki67^+^) and apoptosis (TUNEL^+^) in patient (liver fibrosis stage 0 and 4)-derived hICOs on Ctrl, ORM2, CM from Ctrl human MoMF and ORM2-treated human MoMF was illustrated in fluorescent staining and (**J**) quantitative analyses. (**K**) Gene expressions of *MKI67*, *CCNE1*, *CDKN1A*, *TJP1*, *CLDN1*, *OCLN* and *ORM2* in patient (liver fibrosis stage 0 and 4)-derived hICOs on Ctrl, ORM2, CM from Ctrl human MoMF and ORM2-treated human MoMF were measured (vs Ctrl). Sample sizes: n=3 (technical replicates) per group. CM, conditioned medium; F0/4, fibrosis stage 0/4; hICO, human intrahepatic cholangiocyte organoids; mBEC, mouse biliary epithelial cell; mLMF, mouse liver macrophages (-derived conditioned medium); MoMF, monocyte-derived macrophages (-derived conditioned medium); Od, organoid-derived; ORM2, orosomucoid 2; TUNEL, Terminal Deoxynucleotidyl Transferase-Mediated dUTP Nick End Labelling; WT, wild type. One-way analysis of variance followed by Tukey’s multiple comparison tests were performed. ‘*’represents statistical significance in comparison between MoMF-Ctrl versus Ctrl. ‘$’ represents significant differences between MoMF-ORM2 versus MoMF-Ctrl. p<0.05 as indicated or as compared with controls.

As we evidenced substantial effects of epithelial cell-derived ORM2 on mLMF, we next sought to dissect the macrophage-mediated effects (after their ORM2-induced adaptation) on BECs. Thus, CM was harvested from ORM2-stimulated mLMF and applied on *Mdr2*^−/−^ Od-mBECs. First, cell proliferation in Od-mBECs was significantly suppressed by ORM2-treated mLMF CM (vs control, ORM2 and untreated mLMF CM) (figure 9A). In line, untreated mLMF CM (vs control medium) and ORM2-treated mLMF CM (vs mLMF-Ctrl CM) significantly suppressed the fraction of proliferative (Ki67^+^) Od-mBECs (figure 9B,C). Second, ORM2-treated mLMF CM significantly promoted cell apoptosis (Apopxin^+^) (vs mLMF-Ctrl) (figure 9B,C). Third, both mLMF-ORM2 CM and mBMDM-ORM2 CM significantly increased *Orm2* expression in Od-mBECs (vs mLMF/mBMDM-Ctrl) (figure 9D and 13A). Accordingly, mLMF-ORM2 CM increased ORM2 protein production (vs mLMF-Ctrl) in WT mouse primary cholangiocytes (figure 9E,F). Altogether, this data suggests the direct cellular crosstalk between macrophages and BECs, on the one hand increasing local inflammation and on the other hand, serving as a negative feedback loop for ductular cell expansion.

To corroborate the translational relevance of our findings above, we analysed established hICO cultures derived from either healthy (F0) or cirrhotic (F4) livers. Bright-field microscopy did not reveal potent changes in the morphology of the hICOs (figure 9G). In basal conditions, F4 hICOs tended to express higher levels of *MKI67*, *ORM2* and *CCL2* than F0 hICOs, suggesting a more reactive phenotype for the cells isolated from a diseased liver as observed in mouse intrahepatic cholangiocyte organoids (mICOs) (figure 9H and 13B). Similarly to the experiments performed using mouse cells, CM was harvested from ORM2-treated hMoMF and applied to hICOs. Yet, untreated hMoMF CM significantly suppressed cell proliferation (Ki67^+^) in F0 and F4 hICOs while promoting cell apoptosis (Terminal Deoxynucleotidyl Transferase-Mediated dUTP Nick End Labelling (TUNEL)^+^) only in F0 hICOs (vs Ctrl). Moreover, ORM2-treated hMoMF CM further suppressed cell proliferation (Ki67^+^) in F0 hICOs and promoted cell apoptosis (TUNEL^+^) in F4 hICOs (figure 9I–J). Simultaneously, ORM2-treated hMoMF CM significantly downregulate *MKI67* gene expression but upregulate *ORM2* gene expression in both F0 and F4 hICOs (figure 9K and 13C).

**Figure 10:**
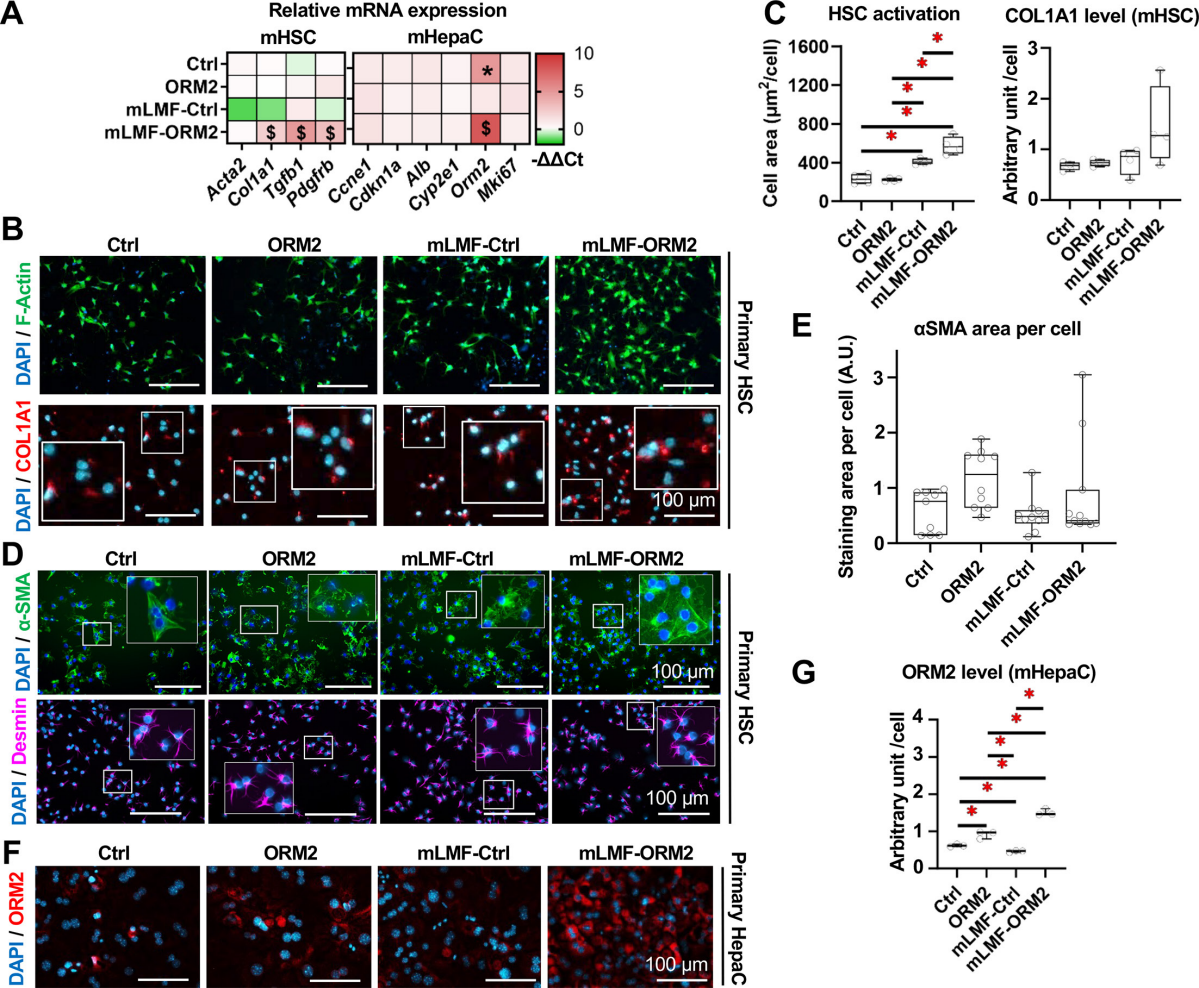
ORM2-activated liver macrophages may promote fibrogenesis in HSCs and ORM2 production in hepatocytes. (**A**) Gene expression of *Acta2*, *Col1a1*, *Tgfb1* and *Pdgfrb* in mouse HSCs and *Ccne1*, *Cdkn1a*, *Alb*, *Cyp2e1*, *Orm2* and *Mki67* in mouse hepatocytes on Ctrl, ORM2, CM from Ctrl mLMF and ORM2-treated mLMF were measured. (**B**) Cell accumulation (F-Actin^+^) and collagen production (COL1A1^+^) in mouse HSCs (cultivated in non-collagen-coated plates) on Ctrl, ORM2, CM from Ctrl mLMF and ORM2-treated mLMF were illustrated by fluorescent staining and (**C**) quantitative analyses. (**D**) Cell identification (Desmin^+^) and activation (α-SMA^+^) in mouse HSCs (cultivated in collagen-coated plates) on Ctrl, ORM2, CM from Ctrl mLMF and ORM2-treated mLMF were illustrated in fluorescent staining and (**E**) quantitative analyses. (**F**) Protein expression of ORM2 in mouse hepatocytes on Ctrl, ORM2, CM from Ctrl mLMF and ORM2-treated mLMF were illustrated in fluorescent staining and (**G**) quantitative analyses. Sample sizes: (**A, C, E and G**) n=4 per group. CM, conditioned medium; COL1A1, collagen type I alpha 1 chain; (m)HepaC, (mouse) hepatocyte; (m)HSC, (mouse) hepatic stellate cell; (m)LMF, (mouse) liver macrophages (-derived conditioned medium); mRNA, messenger RNA; ORM2, orosomucoid 2; α-SMA, alpha smooth muscle actin. One-way analysis of variance followed by Tukey’s multiple comparison tests were performed. ‘*’ represents statistical significance in comparison between mLMF-Ctrl versus Ctrl. ‘$’ represents statistical significance in comparison (mLMF-ORM2 vs mLMF-Ctrl). p<0.05 as indicated or as compared with controls.

To further elucidate crosstalk pathways in this complex cellular niche, we investigated the influence of ORM2-activated LMFs on HSCs and hepatocytes. mLMF-ORM2 CM significantly enhanced the gene expression levels of *Col1a1*, *Tgfb1* and *Pdgfrb* but not *Acta2* in HSCs (figure 10A). Interestingly, the mLMF-Ctrl CM tends to favour HSC activation as evidenced by an increased area occupied by pre-labelled (F-actin^+^) HSCs in culture, an effect that appeared more pronounced when using mLMF-ORM2 CM (vs mLMF-Ctrl) (figure 10B–C). Additionally, mLMF-ORM2 CM treatment for 24 hours demonstrated a non-significant trend towards increased type I collagen immunostaining in those cultures (figure 10B–C). Nonetheless, we did not detect a potent increase in alpha-SMA or desmin immunostaining, although that staining allowed us to validate HSC identity (figure 10D,E). Overall, this data points toward a potential fibrogenic activation of HSC that needs further validation in functional studies. Next, we showed that *Orm2* gene and protein expressions were increased in hepatocytes exposed to ORM2 alone or to mLMF-ORM2 CM (ORM2 vs Ctrl and mLMF-ORM2 vs mLMF-Ctrl) (figure 10A and F–G, online supplemental figure S13D). This data supports the results from multicellular biochip experiments and provides evidence of intricate direct and indirect effects of ORM2 in shaping the biology of the ductular reaction in liver injury. Taken together, our results indicate a bidirectional paracrine ORM2-mediated crosstalk between LMFs and BECs but also to hepatocytes and HSCs, thereby affecting the fate of ductular reaction and, consequently, liver disease progression at a multicellular level.

## Discussion

The biliary niche, or liver portal area, is intensively remodelled during liver disease progression.^2^ These changes include local infiltration of macrophages, fibrogenesis and ductular cell accumulation. Cholangiocyte/BEC-derived ductular cells, formerly regarded as bystanders or mere markers of disease progression, are increasingly valued as active contributors to both physiological and pathogenic processes.^2 50^ Emerging studies have elucidated the remarkable secretory repertoire of BECs, not only in the healthy tissue but also on injuries.^4^ Indeed, injured BECs may progress to apoptosis and cell arrest or obtain proliferative capacities (characteristic of the ductular reaction), which are associated with diverse secretory profiles actively shaping their microenvironment.^4^ Besides representing a largely underexplored area, these phenomena and the present manuscript also illustrate the need for better characterisation and categorisation of the ‘ductular cell’ and ‘BEC’ family, potentially by taking example from the recent progress made in immune cell phenotypic characterisation.

Alongside, bile duct-associated monocyte/macrophage populations are attracting growing attention.^7 51^ Traditional views must be revised to acknowledge that monocytes/macrophages play fundamental roles in not only hepatocyte-associated but also BEC-associated immunological mechanisms.^52 53^ Our study demonstrated that a novel cholangiokine, the acute phase response protein ORM2, can influence the microenvironment of the biliary niche by reprogramming liver macrophages during liver injury. Noticeably, only a few studies have investigated the potential impact of ORM2 on liver disease. A recent study reported that ORM2 directly binds to ITPR2 (a key receptor of the calcium pathway), in turn engaging a calcium-dependent signalling pathway and affecting lipid metabolism processes in hepatocytes.^20^ Here, we report on new ORM2 functions that result from the activation of BEC on ductular reaction and primarily affect liver macrophages. It has been known that calcium pathways regulate fundamental biological processes in cells, including metabolism, secretion profile and cell growth.^54 55^ Expectedly, calcium pathways and the regulation of the intracellular calcium fluxes are crucial in macrophage activation.^56^,^58^ In line, our study identified and characterised for the first time the calcium-mediated liver macrophage polarisation through the ORM2-ITPR2 axis, most notably by enhancing liver macrophage scavenging functions and inflammation-related cytokine release. This BEC-driven macrophage phenotype alteration may open novel research perspectives, particularly since emerging studies have outlined that macrophage phenotypes are significantly altered by metabolic reprogramming, which can be induced by diverse interventions and cytokines.^59^,^62^ Macrophage metabolism (especially lipid metabolism) stands as an essential cellular process that supports membrane biosynthesis and energy storage.^63^,^65^

Moreover, our data suggest that the cellular source rather than the sole presence of ORM2 drives its pathogenic or protective effects when expressed by reactive BECs or hepatocytes, respectively. Intriguingly, despite a high gene expression in basal conditions, *Orm2* silencing in hepatocytes appears to have modest influences on the surrounding microenvironment, in contrast to *Orm2* silencing in reactive BECs. This may be related to unidentified molecular partners co-secreted by ORM2-producing cells, or post-translational modification of ORM2 in different cell types. An alternative explanation is that ORM2 may be stored in resting, healthy hepatocytes and released during inflammation. This may, in turn, potentiate the activation of liver macrophages and reactive BECs and favour disease-promoting inflammation and fibrogenesis. As hepatocytes represent the cells expressing the highest levels of ORM2 gene and protein in the liver, genetic interventions of ORM2 in mouse livers remain challenging particularly for the study of local changes occurring in the biliary niche that are considered as hallmarks of liver diseases. Based on our in-house tailored LoC system,^23^ which we adapted to the present study by introducing BECs, we aimed at modelling the portal multicellular microenvironment for cell-targeted molecular interventions. We are confident this approach will help future studies to unravel previously unrecognised events taking place in the biliary niche and affecting the whole organ, potentially driving new discoveries for understanding orphan cholangiopathies like PSC. Noteworthy, the challenges in acquiring primary human liver cells prompted us to extensively rely on mouse primary cell-based systems, although we were able to validate some key findings in human ICOs and hMoMFs. Differentiated human induced pluripotent stem cells have been suggested as valuable tools for in vitro human liver studies, which exert certain advantages over cell lines, yet the functional relevance of such approaches for the study of the biliary niche remains to be evaluated.^66 67^

In summary, this study demonstrated that on biliary injury, cholangiocyte-derived ORM2 participates in shaping an inflammatory and also, potentially, a fibrogenic biliary niche, serving as a previously unrecognised disease-promoting mechanism. Thus, our study identified novel paracrine signalling pathways between biliary cells and macrophages within the pathogenic ductular reaction, which may provide new targets for intervening with pathogenic responses, while preserving regenerative capacities of an injured liver.

## Supplementary material

10.1136/gutjnl-2024-334425 online supplemental file 1

10.1136/gutjnl-2024-334425 online supplemental file 2

## Data Availability

Data are available in a public, open access repository. Data are available upon reasonable request.
