# Supplementary material for: Reactive cholangiocyte-derived ORM2 drives a pathogenic modulation of the injured biliary niche through macrophage reprogramming
Source: Gut. 2025 Apr 8;74(10):e334425. doi: 10.1136/gutjnl-2024-334425 (PMC12505066; doi:10.1136/gutjnl-2024-334425)
Supplement: online supplemental file 1 [file gutjnl-74-10-s001.docx]

**Supplementary data**

**List of contents**

- **Materials**
- **Methods**
- **Supplementary Figures and Legends**
- **Supplementary Tables**

**Materials**

1. Cell line and organoid-derived (Od) cells

THP-1 (ATCC TIB-202) was purchased from the American Type Culture Collection (Virginia, USA). The purity, contamination, mycoplasma screen and genetic features were assessed and validated by the provider. Further phenotypic characterizations along the course of the project validated cell identity. Cholangiocyte organoids were generated from mouse (wild-type and *Mdr2*^-/-^)-derived primary intrahepatic cholangiocytes (EpCAM^+^). The organoid cultivation method was obtained from the literature [1]. Then, Od-cholangiocytes were obtained by dissociating organoid in domes, and then plated single cells on collagen-coated petri dishes. All animal procedures were approved by LaGeSo Berlin, Germany (Approval numbers: G0243-19, T-CH0028/24 and TCH-020-22).

2. Equipment/supplies

The equipment and supplies that were used in this study are listed below.

| **List of equipment and supplies** |  |  |
| --- | --- | --- |
| **Description** | **Manufacturer** | **Catalog No.** |
| 70-μm strainer | Corning | 352350 |
| 8-well cell culture chamber | Sarstedt | 94.6170.802 |
| AccuSpin 1R Centrifuge: Refrigerated | Thermo Fisher | 4168 |
| Bottle-top filter | Corning | 431161 |
| Bio-Rad Protein Assay Dye Reagent Concentrate | Bio-Rad | 5000006 |
| Falcon tubes - 15 mL | Corning | 352095 |
| Falcon tubes - 50 mL | Corning | 352070 |
| IV catheter | JELCO | 22G IV |
| InkJet Plus Microscope Slides | Thermo Fisher | 12-550-109 |
| Microfluidic biochips | Dynamic42 GmbH | BC002 |
| MS columns | Miltenyi | 130-042-201 |
| Nitrocellulose Membrane | Bio-Rad | 1620146 |
| Perfusion pump and tubing | Ismatec REGLO digital | MS-CA-4/12–100 |
| Protein gel, 12 well, 20 µl | Bio-Rad | 4568125 |
| QuadroMACS Separator | Miltenyi | 130-091-051 |
| Sterican® Standard Cannulas | B Braun | 4657519 |
| Sterile filter for syringe, 0.22 μm | Millipore | SLGP033RB |
| Sterile Petri dishes, 100 mm and 10 mm | Corning | 70165 |
| Sterile Petri dishes, 12-, 24- and 96-well | Costar | 2531 |
| Sterile pipettes - 10 mL | Thermo Fisher | 170356N |
| Sterile pipettes - 25 mL | Thermo Fisher | 170357N |
| Sterile pipettes - 5 mL | Thermo Fisher | 170366N |
| Syringes - 20 mL | BD | 300296 |
| Syringes - 5 mL | BD | 309050 |
| Trans-Blot Turbo Transfer System | Bio-Rad | - |

3. Chemicals and proteins

Chemicals and proteins that were used in this study are listed below.

| **List of chemicals and proteins** |  |  |
| --- | --- | --- |
| **Description** | **Manufacturer** | **Catalog No.** |
| 2-APB | Sigma-Aldrich | 100065 |
| Albumin from bovine serum, BSA | Sigma-Aldrich | A9430 |
| Biliatresone, BT | Axon Medchem | AXON 2867 |
| Blotting Grade Blocker Non-Fat Dry Milk | Bio-Rad | 1706404XTU |
| Calcium chloride dihydrate, CaCl_2_·2H_2_O | Sigma-Aldrich | C7902 |
| Chenodeoxycholic acid, CDCA | Thermo Fisher | C9377 |
| Collagenase type 4 | CellSystems | LS004186 |
| D-(+)-Glucose | Sigma-Aldrich | G8270 |
| DNase I | Roche | 10104159001 |
| EDTA | Sigma-Aldrich | E9884 |
| EGTA | Sigma-Aldrich | E4378 |
| HEPES | Sigma-Aldrich | H4034 |
| Magnesium chloride hexahydrate, MgCl_2_·6H_2_O | Sigma-Aldrich | 930970 |
| Magnesium sulfate heptahydrate, MgSO_4_·7H_2_O | Sigma-Aldrich | 230391 |
| Methanol | Sigma-Aldrich | 34860 |
| Nycodenz | Accurate Chemical | 1002424 |
| Oleic acid, OA | Sigma-Aldrich | O1008 |
| Palmitic acid, PA | Sigma-Aldrich | P0500 |
| Paracetamol, APAP | Sigma-Aldrich | BP371 |
| Paraformaldehyde, PFA | Sigma-Aldrich | 158127 |
| Pierce Protease and Phosphatase Inhibitor Mini Tablets, EDTA- free | Thermo fisher | A32961 |
| Phorbol 12-myristate 13-acetate, PMA | Sigma-Aldrich | P8139 |
| Potassium chloride, KCl | Sigma-Aldrich | P9333 |
| Potassium phosphate monobasic, KH_2_PO_4_ | Sigma-Aldrich | 60229 |
| Protease | Sigma-Aldrich | P5147 |
| Sodium bicarbonate, NaHCO3 | Sigma-Aldrich | S5761 |
| Sodium chloride, NaCl | Sigma-Aldrich | S3014 |
| Sodium Phosphate monobasic monohydrate, NaH2PO4·H2O | Thermo Fisher | S369 |
| Sodium phosphate, Na2HPO4 | Sigma-Aldrich | S9763 |
| Human recombinant ORM2 | Bio-mol | 156190.5 |
| Mouse recombinant ORM2 | BIZOL | LS-G14265-50 |

4. Solutions

4.1. Solutions (pre-made)

Commercialized solutions that were used in this study are listed below**.**

| **List of solutions** |  |  |
| --- | --- | --- |
| **Description** | **Manufacturer** | **Catalog No.** |
| Acetic acid solution, CH3CO2H | Sigma-Aldrich | 45754 |
| BD Pharm Lyse™ Lysing Buffer (10×) | BD | 555899 |
| Citrate buffer antigen retrieval | Thermo Fisher | AP-9003-500 |
| Collagen I, rat tail | Thermo Fisher | A1048301 |
| Cultrex Organoid Harvesting Solution | Bio-Techne GmbH | 3700-100-01 |
| Dulbecco's phosphate-buffered saline, DPBS | Gibco | 14190144 |
| ECL Substrate | Bio-Rad | MABN92 |
| Ethanol solution 70 % | Thermo Fisher | 15542393 |
| Fetal bovine serum, FBS | Gibco | A4736201 |
| Hanks' Balanced Salt Solution, HBSS | Gibco | 24020117 |
| Image-iT FX signal enhancer | Thermo Fisher | I36933 |
| Isoflurane | CP-pharma | 798-932 |
| Lipofectamine™ RNAiMAX | Thermo Fisher | 13778100 |
| Lipopolysaccharide (LPS) Solution (500X) | Sigma-Aldrich | 00-4976-93 |
| Lysing buffer (10×) | BioLegend | 420301 |
| Normal goat serum, NGS (10%) | Thermo Fisher | 50062Z |
| PBS-10× | Life Technologies | 10010-23 |
| Percoll-100% | Sigma-Aldrich | P1644 |
| Phosphate-buffered saline, PBS | Gibco | 70011044 |
| Primary hepatocyte maintenance supplements | Gibco | CM4000 |
| RIPA-Lysepuffer, 10x | Sigma-Aldrich | 20-188 |
| 10x Tris Buffered Saline (TBS) | Bio-Rad | 1706435 |
| Tris-EDTA buffer-10×, pH9.0 | Novus Biologicals | NB900-62085 |
| Triton X-100 solution | Sigma-Aldrich | 93443 |
| Tween-20 | Roth | 9127.1 |
| Universal SYBR Green Fast qPCR Mix | ABclonal | RK21203 |
| VectaMount AQ aqueous mounting medium | Vector | H-5501 |
| William’s E medium | Gibco | W1878-500ML |

4.2. Solutions (home-made)

Protocols for preparing the solutions or buffers used in this study are described below.

1. EGTA buffer: Prepare the solution by dissolving the components of the recipe given below in 1 L of ddH_2_O. Adjust the pH to 7.4 and filter the solution through a 0.2-μm bottle-top filter. The constituted solution can be stored at 4°C for up to 6 months.

**Composition of the EGTA buffer.**

| **Reagent** | **Final concentration (mg/L)** |
| --- | --- |
| NaCl | 8,000 |
| KCl | 400 |
| NaH_2_PO_4_·H_2_O | 88.17 |
| Na_2_HPO_4_ | 120.45 |
| HEPES | 2,380 |
| NaHCO_3_ | 350 |
| EGTA | 190 |
| D-(+)-Glucose | 900 |

1. Enzyme buffer: Prepare the solution by dissolving the components of the recipe given below in 1 L of ddH_2_O. Adjust the pH to 7.4 and filter the solution through a 0.2-μm bottle-top filter. The ready-to-use solution can be stored at 4°C for up to 6 months.

**Composition of the liver digestion buffer**

| **Reagent** | **Final concentration (mg/L)** |
| --- | --- |
| NaCl | 8,000 |
| KCl | 400 |
| NaH_2_PO_4_·H_2_O | 88.17 |
| Na_2_HPO_4_ | 120.45 |
| HEPES | 2,380 |
| NaHCO_3_ | 350 |
| CaCl_2_·2H_2_O | 560 |

1. GBSS/A buffer: Prepare the solution by dissolving the components of the recipe given below in 1 L of ddH_2_O. Adjust the pH to 7.4 and filter the solution through a 0.2-μm bottle-top filter. The constituted solution can be stored at 4°C for up to 6 months.

**Composition of the GBSS/A buffer**

| **Reagent** | **Final concentration (mg/L)** |
| --- | --- |
| KCl | 370 |
| MgCl_2_·6H_2_O | 210 |
| MgSO_4_·7H_2_O | 70 |
| Na_2_HPO_4_ | 59.6 |
| KH_2_PO_4_ | 30 |
| Glucose | 991 |
| NaHCO_3_ | 227 |
| CaCl_2_·2H_2_O | 225 |

1. GBSS/B buffer: Add 8 g/L NaCl into 1 L of GBSS/A buffer as described above to make GBSS/B buffer. Adjust the pH to 7.35 – 7.4 and filter the solution through a 0.2-μm bottle-top filter. The constituted solution can be stored at 4°C for up to 6 months.
2. Digestion buffers: Prepare the solution by dissolving the components of the recipe given below. The buffers need to be freshly made for each experiment. Filter the buffer with a 0.22-μm filter before use.
   1. Digestion buffer I: Dissolve 4.4 U collagenase type 4 in 50 mL enzyme buffer.
   2. Digestion buffer II: Dissolve 4.4U collagenase type 4, 40 µg DNase I and 4.5 mg pronase in 50 mL enzyme buffer.
3. DNase I solution: Dissolve 100 mg in 100 mL PBS to make 1 mg/mL solution.
4. Percoll-50% solution: Dissolve 10.8 mL Percoll-100% and 1.2 mL 10X-PBS in 14.5 mL 1×-PBS to make Percoll-50% solution. Mix it thoroughly.
5. Nycodenz solution: Dissolve 4.94 g nycodenz in 15ml GBSS/A buffer and filter it through a 0.22-μm filter. Adjust the solution volume to 17ml. Optional: Add phenol red to indicate gradient layers.
6. Magnetic-activated cell sorting (MACS) buffer: Dissolve 250 μg BSA and 37.2 μg EDTA in 50 mL DPBS. Filter it through a 0.22-μm filter. The constituted solution can be stored at 4°C for up to 2 weeks.
7. Collagen-coating buffer: Dissolve 660 µL collagen-I in 50 mL DPBS containing 0.12% acetic acid. Filter it through a 0.22-μm filter.
8. Blocking buffer: 5% normal goat serum and 0.3% Triton-X in DPBS.
9. Antibody buffer: 1% BSA and 0.3% Triton X in DPBS.
10. FFA solution: Dissolve PA or OA in DPBS containing 1% BSA - the ratios of PA and OA in the FFA mixture can be adjusted for different purposes. The final working FFA concentration is 30 mM.
11. Blocking buffer: Respectively dilute NGS and TritonX-100 into the DPBS to make 5% NGS and 0.3% Triton X-100 as final concentrations.
12. Antibody buffer: Respectively dilute BSA and TritonX-100 into the DPBS to make 1% BSA and 0.3% Triton X-100 as final concentrations.

**Methods**

1. Primary cell isolation and cultivation

1.1. Mouse liver perfusion and cell isolation

Livers collected from wild-type (WT) or Mdr2^-/-^ mice were perfused and digested. Primary hepatocytes, bilary epithelial cells (BECs), hepatic stellate cells (HSCs), Kupffer cells (KCs) and liver endothelial sinusoidal cells (LSECs) were isolated using centrifugation and MACS. Circulating immune cells (CICs) were isolated from fresh mouse or human blood. Detailed protocols have been described in our publication [2].

1.2. Mouse monocyte-derived macrophage (MoMF) isolation and differentiation

Femurs with muscle and fascia completely removed were collected from sacrificed mice and rinsed in cold DPBS buffer. Remove both ends of the femurs and flush out the bone marrow with cell culture medium (William’s E medium + 10% FBS + 1% penicillin/streptomycin). Cell suspensions were seeded in petri dishes after going through a 70-μm cell strainer. Bone marrow cells were treated with 20 ng/mL macrophage colony-stimulating factor (M-CSF) for 7 to 10 days to generate macrophage-like phenotypes.

1.3. Mouse cholangiocyte organoid culture and dissociation

WT and Mdr2^-/-^ mouse intrahepatic cholangiocytes were isolated using the MACS approach with mouse EpCAM beads. Cholangiocyte organoids were cultivated following the well-established protocol [3]. Organoids were dissociated into single cells. Then, cells were seeded in collagen-coated plates with regular cell culture medium and incubated at 37℃ 5% CO^2^. Mouse cholangiocyte organoids were dissociated by adding cultrex organoid harvesting solution (Bio-Techne GmbH, Germany) in to the matrigel. After 30 min incubation on ice, organoids were dissociated to single cell suspension and ready to be seeded on collagen-coated petri dishes or biochips.

2. Cell treatment

2.1. THP-1 cell activation

Cells were treated with 50 ng/mL PMA for 2 days to achieve immature macrophages (unpolarized). The activation status was assessed by morphological and attaching features.

2.2. Cell injury induction

Mouse primary cholangiocytes and hepatocytes were treated with 2 µg/mL biliatresone, 1 mM CDCA, 25 mM APAP or 300 µM free fatty acids (FFAs, oleic acid:palmitic acid = 1:1) for 24 hours.

2.3. Macrophage stimulation

THP-1 derived macrophages, human MoMFs (from healthy lab volunteers), mouse MOMFs and mouse liver macrophages were respectively treated with 1 μg/mL ORM2 (or 100 ng/mL LPS) for 24 hours. 75 μM 2-APB was introduced to mouse primary liver macrophages to suppress intracellular calcium concentration.

3. Biliary -niche-on-a-chip (BoC) and Liver-on-a-chip (LoC) experiments

1) Sterilize the surface and inner cavities of the biochip by pipetting in 70% ethanol and incubating for 40 min.

2) Wash the cavities 3 times with DPBS buffer.

3) Pipette 500 mL collagen coating buffer in each cavity and incubate for 15 min.

4) Wash the cavities 3 times with DPBS buffer. Fill each cavity with 500 μL medium. Block connections between the cavities with plugs.

5) Cell seeding and intervention strategies:

a) On Day 1, seed 300,000 organoid-derived cholangiocytes in the upper cavity.

b) On Day 2, silence Orm2 expression of cholangiocytes in the BoC (following the methods in Section 3.2.4).

c) On Day 3, seed 300,000 LSECs and 100,000 liver macrophages in the lower cavity. Incubate overnight at 37°C, 5% CO_2_.

d) On Day 4, flip back the chip. Seed 200,000 HSCs in the upper cavity. Incubate at 37°C, 5% CO2 for 48 hours (until Day 6).

6) Bio-chip perfusion strategies (on Day 6):

a) Connect the tubing to each lower cavity. Add 1 mL medium containing 100,000 CICs into each reservoir.

b) Start perfusion through the pumping channels at 50 µL / min.

c) Transfer chip-pump assemblies into the incubator. Incubate at 37°C, 5% CO2 for 30 min (from 30 min to 48 hours depending on the experimental needs).

CAUTION: Bubbles should be avoided during cell seeding into the biochip, medium changing and CIC perfusion.

7) Sampling strategies:

a) Apply live cell dye on cells and observe under microscope.

b) Harvest the perfusion medium and centrifuge at 4°C, 400 × g for 10 min. Collect CICs in the cell pellets. Keep the supernatant for further analyses.

c) Cut off membranes to harvest resident cells for RNA extraction, or for immunohistochemistry (IHC) detection after fixation with 4% PFA.

LoC exprimental strategies have been described in our publication [2].

4. Tissue staining

Sequential multiplex immunohistochemistry (mIHC) was performed as previously described [4-6]. The antibody elution buffer was prepared by mixing 675 μL distilled water, 125 μL 0.5 M Tris-HCl pH 6.8, 200 μL 10% (w/v) sodium dodecyl sulfate, and 8 μL 2-mercaptoethanol. The list of antibodies is provided in Supplementary Table 1. Image analysis was performed with an in-house optimized image processing pipeline and software tools [4]. The antibody elution buffer was prepared by mixing 675 μL distilled water, 125 μL 0.5 M Tris-HCl pH 6.8, 200 μL 10% (w/v) sodium dodecyl sulfate, and 8 μL 2-mercaptoethanol. The list of antibodies is provided in Supplementary Table 1.

5. Live cell staining

Viable adherent cells in petri dishes or chamber slides were stained with diverse live cell dyes or kits for different purposes: (1) Cells were stained with Hoechst 33342 to indicate the nucleus. (2) Cells were stained with BODIPY dye to illustrate intracellular lipid droplets. (3) Cells were stained with a CellMask plasma membrane stain kit to depict the cell membrane. (4) Cells were stained with an Apoptosis/ Necrosis Assay Kit to assess cell apoptosis, cell death and viability. Stained live cells can be immediately observed and traced under a fluorescent microscope. The list of antibodies is provided in Supplementary Table 1.

6. Immunocytochemistry (ICC)

1) Fixation buffer preparation: Dissolve 2 g paraformaldehyde (PFA) in 50 mL 1×-PBS. Put in a water bath (70 – 98 ℃) for 40 min. Store at 4 ℃ for 2 weeks.

2) Cell fixation: Wash cells with PBS. Pre-warm 2% PFA at “37 ℃ and overlay them on each well (or re-suspend cell pellet). Incubate at room temperature for 15 min under a hood. Rinse cells with PBS before staining.

3) IHC staining: Block in blocking buffer (PBS + 5% normal goat serum + 0.3% Triton-X) for 50 min at room temperature. Without washing, apply primary antibodies diluted (1:200) in antibody buffer (PBS + 1% BSA + 0.3% Triton-X). Incubate with primary antibodies at room temperature for 4h or overnight. Rinse 3 times with PBS. Apply secondary antibodies diluted (around 1:1000) in antibody buffer (PBS + 1% BSA + 0.3% Triton-X). Incubate with secondary antibodies for 30 min at room temperature. Rinse 3 times in PBS. Apply DAPI solution in PBS, incubate at room temperature for 10 min. Rinse slides 3 times in PBS.

4) For cellular senescence detection: Cells are fixed in 2% PFA for 10 min, and then processed using a Senescence detection kit, following the manufacturers’ instructions. Stained cells can be observed under a fluorescent microscope.

The list of antibodies is provided in Supplementary Table 1.

7. Microscopy and western blot image processing and analysis

Images derived from western blot, immunohistochemistry and immunocytochemistry were processed and analyzed using Image J 1.53q (NIH, USA). Multiplex immunofluorescence images were analyzed as previously described [2, 4, 6]. Briefly, large scan tiled raw .CZI files acquired from a ZEISS Observer7 microscope were stitched using a shading reference control image. Images were then exported as .TIF, registered using the DAPI signal to allow for consecutive days of imaging alignment. Images displayed in the main and supplementary figures have been subjected to background subtraction using a rolling ball algorithm, except for ORM2 and ITPR2 images. CellSeg [7, 8] and Ilastik v1.3.2 [9] were used for nuclei and cell segmentation, respectively. Imaging cytometry was performed in CellProfiler v4.2.5 [10]. For staining intensity measurements, single channel raw images (without background subtraction) were used. Relative ORM2 staining visuals were generated by applying the default phase LUT to the raw grayscale fluorescence images in FIJI.

8. Bioinformatics - transcriptomic datasets analysis

Bulk RNA sequencing analysis was performed by the genomics platform from Berliner Institut für Medizinische Systembiologie des Max Delbrück Center (MDC-BIMSB). To extract meaningful biological insights from the raw sequencing data, we used a suite of R packages tailored for different stages of the analysis pipeline. The initial quality control and preprocessing steps were performed using the ‘BiocParallel (version 1.40.0)’ and ‘ShortRead (version 1.64.0)’ packages, which allow for efficient parallel processing and effective handling of raw sequence reads. Subsequent alignment and quantification of transcript abundance was performed using the ‘edgeR (4.4.2)’ packages, facilitating differential expression analysis and identification of significantly modulated genes[11]. Functional enrichment analysis was performed using the Gene Ontology (GO) and Kyoto Encyclopedia of Genes and Genomes (KEGG) databases [12, 13]. Cell type enrichment analysis was performed using the ‘Xcell (version 2.0)’ package [14]. Secreted factors were identified accoding to the Human Protein Atlas database [15]. Published datasets were obtained from the Gene Expression Omnibus (GEO) database (<https://www.ncbi.nlm.nih.gov/gds>). Published bulk or single-cell/single-nuclei datasets were obtained from GepLiver [16] and GEO database [17-30]. The secretome dataset was obtained from Human Protein Atlas (https://www.proteinatlas.org/).

9. Flow cytometry for immune cell characterization

Human and mouse macrophages were collected and cells were centrifuged at 400 x g for 5 minutes. The cells were then incubated with a fixable viability dye (Zombie NIR™ Fixable Viability Kit; Biolegend, USA) at a 1:5000 dilution for 10 minutes at 4°C, followed by an incubation with fluorochrome-conjugated antibodies (Supplementary Table 1) in blocking buffer (PBS + 2% BSA + 2% normal mouse/rat/rabbit/human serum) for 20 minutes at 4°C. Cells were fixed with PBS containing 1% formalin for 10 minutes at 4°C. Finally, the cells were resuspended in 200 µL PBS and 10 µL counting beads (106 beads / mL) were added to each sample. Multispectral flow cytometry was performed using the Cytek® Aurora.

10. Cytokine and transaminase measurements

Supernatant samples were collected from cell culture and centrifuged at 1000 x g for 10 minutes at 4°C. The resulting supernatant samples were stored at -80°C until analysis. Aspartate aminotransferase (AST) and alanine transaminase (ALT) levels were measured by Labor Berlin – Charité Vivantes GmbH, Berlin, Germany, using standard procedures. Cytokine levels were measured using the LEGENDplex mouse inflammation panel (Biolegend, USA) according to the manufacturer’s manuals (Supplementary Table 1). BD FACSCanto™ Ⅱ set up in the PE and APC channels was used to perform the measurements according to the manufacturer’s manuals.

11. RNA isolation and RT-qPCR measurements

Total RNA samples were extracted from the cells using the RNeasy Kit (Qiagen, Germany) according to the manufacturer’s instructions. Quantitative real-time PCR was carried out using the Applied Biosystems Real-Time PCR System (ThermoFisher, USA) and SYBR RT-PCR kits (Roche, Switzerland). The -ΔΔCycle threshold (Ct) analysis method was used to assess relative mRNA expression normalized to *18S*. Primer sequences are listed in Supplementary Table 2.

12. Gene expression interference assay

Mouse primary liver macrophages and cholangiocytes (primary and organoid-derived) were seeded 12 – 24 hours before gene expression interference assay in a serum-reduced cell culture medium (William’s E medium + 1% fetal bovine serum + 1% penicillin/streptomycin). The mixture of 10 pmol small-interfering RNA (siRNA) (target or control) and 1 μL Lipofectamine™ RNAiMAX reagent was made for every 100 μL of serum-free William’s E medium, and incubated at room temperature for 30 min. Then, the siRNA mixture was introduced to the cells and replaced with regular cell culture medium after 4 – 6 hours of incubation at 37℃, 5% CO_2_. More technical details can be found in the manufacturer’s instructions (Thermo Fisher, U.S.A.).

13. Cellular calcium measurement

Fluo-8 Calcium Flux Assay Kit (ab112129) was purchased from Abcam. Cultivated mouse liver macrophages were stained by the Fluo-8 fluorescent dye. The fluorescence intensity was measured by a microplate reader (ThermoFisher, USA) to assess intracellular calcium mobilization in cells. The experiments were conducted following manufacturer’s instruction.

14. Western blot for protein quantification

For protein extraction, cells were lysed in RIPA lysis buffer and centrifuged at 15,000 × g for 10 min. Supernatant from cell lysate was transferred to a new tube and protein concentration was determined using Bradford protein assay. The protein extract was mixed with the Laemmli buffer for SDS-PAGE gel electrophoresis, then transferred onto nitrocellulose membrane. Membranes were blocked with 5% non-fat dry milk for 1 h at room temperature and incubated with the specific first antibodies overnight at 4°C. After horseradish peroxidase-conjugated secondary antibody (1:5000) incubation or after HRP-conjugated β-Actin incubation chemiluminescent signals were detected by ChemiDoc MP Imaging System (BIO-RAD) after exposure to enhanced chemiluminescence substrate (Clarity Western ECL Substrate, BIO-RAD). Signal intensity was quantified using Image J software. Antibody against CD36 (1:1000) was purchased from Cell Signaling Technology and antibody against β-actin (1:25000) was purchased from Abcam. Protein levels were normalized to the level of β-actin.

**Supplementary references**

1. Tysoe, O.C., A.W. Justin, T. Brevini, S.E. Chen, K.T. Mahbubani, A.K. Frank, H. Zedira, E. Melum, K. Saeb-Parsy, A.E. Markaki, L. Vallier, and F. Sampaziotis, *Isolation and propagation of primary human cholangiocyte organoids for the generation of bioengineered biliary tissue.* Nat Protoc, 2019. **14**(6): p. 1884-1925.

2. Liu, H., G. Yin, M.S. Kohlhepp, F. Schumacher, J. Hundertmark, M.I.A. Hassan, F. Heymann, T. Puengel, B. Kleuser, A.S. Mosig, F. Tacke, and A. Guillot, *Dissecting Acute Drug-Induced Hepatotoxicity and Therapeutic Responses of Steatotic Liver Disease Using Primary Mouse Liver and Blood Cells in a Liver-On-A-Chip Model.* Adv Sci (Weinh), 2024. **11**(30): p. e2403516.

3. Broutier, L., A. Andersson-Rolf, C.J. Hindley, S.F. Boj, H. Clevers, B.K. Koo, and M. Huch, *Culture and establishment of self-renewing human and mouse adult liver and pancreas 3D organoids and their genetic manipulation.* Nat Protoc, 2016. **11**(9): p. 1724-43.

4. Guillot, A., M.S. Kohlhepp, A. Bruneau, F. Heymann, and F. Tacke, *Deciphering the Immune Microenvironment on A Single Archival Formalin-Fixed Paraffin-Embedded Tissue Section by An Immediately Implementable Multiplex Fluorescence Immunostaining Protocol.* Cancers (Basel), 2020. **12**(9).

5. Guillot, A., M.S. Kohlhepp, and F. Tacke, *Multiplex Immunostaining to Spatially Resolve the Cellular Landscape in Human and Mouse Livers.* Methods Mol Biol, 2023. **2669**: p. 245-255.

6. Guillot, A., M. Winkler, M. Silva Afonso, A. Aggarwal, D. Lopez, H. Berger, M.S. Kohlhepp, H. Liu, B. Ozdirik, J. Eschrich, J. Ma, M. Peiseler, F. Heymann, S. Pendem, S. Mahadevan, B. Gao, L. Diehl, R. Gupta, and F. Tacke, *Mapping the hepatic immune landscape identifies monocytic macrophages as key drivers of steatohepatitis and cholangiopathy progression.* Hepatology, 2023. **78**(1): p. 150-166.

7. Lee, M.Y., J.S. Bedia, S.S. Bhate, G.L. Barlow, D. Phillips, W.J. Fantl, G.P. Nolan, and C.M. Schurch, *CellSeg: a robust, pre-trained nucleus segmentation and pixel quantification software for highly multiplexed fluorescence images.* BMC Bioinformatics, 2022. **23**(1): p. 46.

8. Sternberg, *Biomedical Image Processing.* Computer, 1983. **16**(1): p. 22-34.

9. Berg, S., D. Kutra, T. Kroeger, C.N. Straehle, B.X. Kausler, C. Haubold, M. Schiegg, J. Ales, T. Beier, M. Rudy, K. Eren, J.I. Cervantes, B. Xu, F. Beuttenmueller, A. Wolny, C. Zhang, U. Koethe, F.A. Hamprecht, and A. Kreshuk, *ilastik: interactive machine learning for (bio)image analysis.* Nat Methods, 2019. **16**(12): p. 1226-1232.

10. Stirling, D.R., M.J. Swain-Bowden, A.M. Lucas, A.E. Carpenter, B.A. Cimini, and A. Goodman, *CellProfiler 4: improvements in speed, utility and usability.* BMC Bioinformatics, 2021. **22**(1): p. 433.

11. Robinson, M.D., D.J. McCarthy, and G.K. Smyth, *edgeR: a Bioconductor package for differential expression analysis of digital gene expression data.* Bioinformatics, 2010. **26**(1): p. 139-40.

12. Gene Ontology, C., *Gene Ontology Consortium: going forward.* Nucleic Acids Res, 2015. **43**(Database issue): p. D1049-56.

13. Kanehisa, M. and S. Goto, *KEGG: kyoto encyclopedia of genes and genomes.* Nucleic Acids Res, 2000. **28**(1): p. 27-30.

14. Aran, D., Z. Hu, and A.J. Butte, *xCell: digitally portraying the tissue cellular heterogeneity landscape.* Genome Biol, 2017. **18**(1): p. 220.

15. Thul, P.J. and C. Lindskog, *The human protein atlas: A spatial map of the human proteome.* Protein Sci, 2018. **27**(1): p. 233-244.

16. Li, Z., H. Zhang, Q. Li, W. Feng, X. Jia, R. Zhou, Y. Huang, Y. Li, Z. Hu, X. Hu, X. Zhu, and S. Huang, *GepLiver: an integrative liver expression atlas spanning developmental stages and liver disease phases.* Sci Data, 2023. **10**(1): p. 376.

17. Guillot, A., L. Guerri, D. Feng, S.J. Kim, Y.A. Ahmed, J. Paloczi, Y. He, K. Schuebel, S. Dai, F. Liu, P. Pacher, T. Kisseleva, X. Qin, D. Goldman, F. Tacke, and B. Gao, *Bile acid-activated macrophages promote biliary epithelial cell proliferation through integrin alphavbeta6 upregulation following liver injury.* J Clin Invest, 2021. **131**(9).

18. Kennedy, L., G. Carpino, T. Owen, L. Ceci, D. Kundu, V. Meadows, K. Kyritsi, A. Franchitto, P. Onori, A. Isidan, W. Zhang, B. Ekser, D. Alvaro, E. Gaudio, M.E. Gershwin, H. Francis, S. Glaser, and G. Alpini, *Secretin alleviates biliary and liver injury during late-stage primary biliary cholangitis via restoration of secretory processes.* J Hepatol, 2023. **78**(1): p. 99-113.

19. Yildiz, E., G. El Alam, A. Perino, A. Jalil, P.D. Denechaud, K. Huber, L. Fajas, J. Auwerx, G. Sorrentino, and K. Schoonjans, *Hepatic lipid overload triggers biliary epithelial cell activation via E2Fs.* Elife, 2023. **12**.

20. Xu, Y.F., Y. Yao, M. Ma, S.H. Yang, P. Jiang, J. Wang, K. Tsuneyama, C. Wang, X. Liu, L. Li, and Z.X. Lian, *The Proinflammatory Cytokines IL-18, IL-21, and IFN-gamma Differentially Regulate Liver Inflammation and Anti-Mitochondrial Antibody Level in a Murine Model of Primary Biliary Cholangitis.* J Immunol Res, 2022. **2022**: p. 7111445.

21. Wang, H., B.P. Vohra, Y. Zhang, and R.O. Heuckeroth, *Transcriptional profiling after bile duct ligation identifies PAI-1 as a contributor to cholestatic injury in mice.* Hepatology, 2005. **42**(5): p. 1099-108.

22. Paillet, J., C. Plantureux, S. Levesque, J. Le Naour, G. Stoll, A. Sauvat, P. Caudana, J. Tosello Boari, N. Bloy, S. Lachkar, I. Martins, P. Opolon, A. Checcoli, A. Delaune, N. Robil, P. de la Grange, J. Hamroune, F. Letourneur, G. Autret, P.S.C. Leung, M.E. Gershwin, J.S. Zhu, M.J. Kurth, B. Lekbaby, J. Augustin, Y. Kim, S. Gujar, C. Coulouarn, L. Fouassier, L. Zitvogel, E. Piaggio, C. Housset, P. Soussan, M.C. Maiuri, G. Kroemer, and J.G. Pol, *Autoimmunity affecting the biliary tract fuels the immunosurveillance of cholangiocarcinoma.* J Exp Med, 2021. **218**(10).

23. Lei, L., A. Bruneau, H. El Mourabit, J. Guegan, T. Folseraas, S. Lemoinne, T.H. Karlsen, B. Hoareau, R. Morichon, E. Gonzalez-Sanchez, C. Goumard, V. Ratziu, P. Charbord, J. Gautheron, F. Tacke, T. Jaffredo, A. Cadoret, and C. Housset, *Portal fibroblasts with mesenchymal stem cell features form a reservoir of proliferative myofibroblasts in liver fibrosis.* Hepatology, 2022. **76**(5): p. 1360-1375.

24. Frades, I., E. Andreasson, J.M. Mato, E. Alexandersson, R. Matthiesen, and M.L. Martinez-Chantar, *Integrative genomic signatures of hepatocellular carcinoma derived from nonalcoholic Fatty liver disease.* PLoS One, 2015. **10**(5): p. e0124544.

25. Chen, Y.M., C.F. Lian, Q.W. Sun, T.T. Wang, Y.Y. Liu, J. Ye, L.L. Gao, Y.F. Yang, S.N. Liu, Z.F. Shen, and Y.L. Liu, *Ramulus Mori (Sangzhi) Alkaloids Alleviate High-Fat Diet-Induced Obesity and Nonalcoholic Fatty Liver Disease in Mice.* Antioxidants (Basel), 2022. **11**(5).

26. Ahrens, M., O. Ammerpohl, W. von Schonfels, J. Kolarova, S. Bens, T. Itzel, A. Teufel, A. Herrmann, M. Brosch, H. Hinrichsen, W. Erhart, J. Egberts, B. Sipos, S. Schreiber, R. Hasler, F. Stickel, T. Becker, M. Krawczak, C. Rocken, R. Siebert, C. Schafmayer, and J. Hampe, *DNA methylation analysis in nonalcoholic fatty liver disease suggests distinct disease-specific and remodeling signatures after bariatric surgery.* Cell Metab, 2013. **18**(2): p. 296-302.

27. Andrews, T.S., D. Nakib, C.T. Perciani, X.Z. Ma, L. Liu, E. Winter, D. Camat, S.W. Chung, P. Lumanto, J. Manuel, S. Mangroo, B. Hansen, B. Arpinder, C. Thoeni, B. Sayed, J. Feld, A. Gehring, A. Gulamhusein, G.M. Hirschfield, A. Ricciuto, G.D. Bader, I.D. McGilvray, and S. MacParland, *Single-cell, single-nucleus, and spatial transcriptomics characterization of the immunological landscape in the healthy and PSC human liver.* J Hepatol, 2024. **80**(5): p. 730-743.

28. Pinyol, R., S. Torrecilla, H. Wang, C. Montironi, M. Pique-Gili, M. Torres-Martin, L. Wei-Qiang, C.E. Willoughby, P. Ramadori, C. Andreu-Oller, P. Taik, Y.A. Lee, A. Moeini, J. Peix, S. Faure-Dupuy, T. Riedl, S. Schuehle, C.P. Oliveira, V.A. Alves, P. Boffetta, A. Lachenmayer, S. Roessler, B. Minguez, P. Schirmacher, J.F. Dufour, S.N. Thung, H.L. Reeves, F.J. Carrilho, C. Chang, A.V. Uzilov, M. Heikenwalder, A. Sanyal, S.L. Friedman, D. Sia, and J.M. Llovet, *Molecular characterisation of hepatocellular carcinoma in patients with non-alcoholic steatohepatitis.* J Hepatol, 2021. **75**(4): p. 865-878.

29. Murphy, S.K., H. Yang, C.A. Moylan, H. Pang, A. Dellinger, M.F. Abdelmalek, M.E. Garrett, A. Ashley-Koch, A. Suzuki, H.L. Tillmann, M.A. Hauser, and A.M. Diehl, *Relationship between methylome and transcriptome in patients with nonalcoholic fatty liver disease.* Gastroenterology, 2013. **145**(5): p. 1076-87.

30. Moylan, C.A., H. Pang, A. Dellinger, A. Suzuki, M.E. Garrett, C.D. Guy, S.K. Murphy, A.E. Ashley-Koch, S.S. Choi, G.A. Michelotti, D.D. Hampton, Y. Chen, H.L. Tillmann, M.A. Hauser, M.F. Abdelmalek, and A.M. Diehl, *Hepatic gene expression profiles differentiate presymptomatic patients with mild versus severe nonalcoholic fatty liver disease.* Hepatology, 2014. **59**(2): p. 471-82.

**Supplementary Figures and Legends**


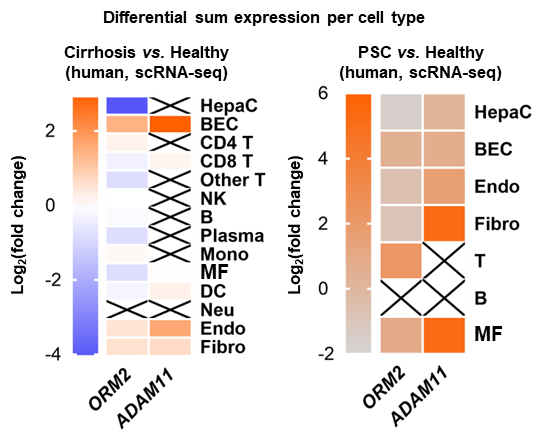


**Figure S1.** Gene expression of *ORM2* and *ADAM11* in human liver cells. Sum (total) gene expression of *ORM2* and *ADAM11* in diverse liver cells from healthy donors and patients with liver cirrhosis and PSC were illustrated in heatmaps. scRNA-seq data was obtained from GepLiver database and GEO datasets (ID: GSE247128 and GSE243977). X’ in the block represents undetectable data. Abbreviations: B: B lymphocytes; BEC: biliary epithelial cells; DC: dendritic cells; Endo: endothelial cells; Fibro: fibroblasts; HepaC: hepatocytes; MASLD: metabolic dysfunction-associated steatotic liver disease; Mono: monocytes; MF: macrophages; Neu: neutrophils; NK: natural killer cells; Plasma: plasma cells; PSC: primary sclerosing cholangitis; T: T lymphocytes.


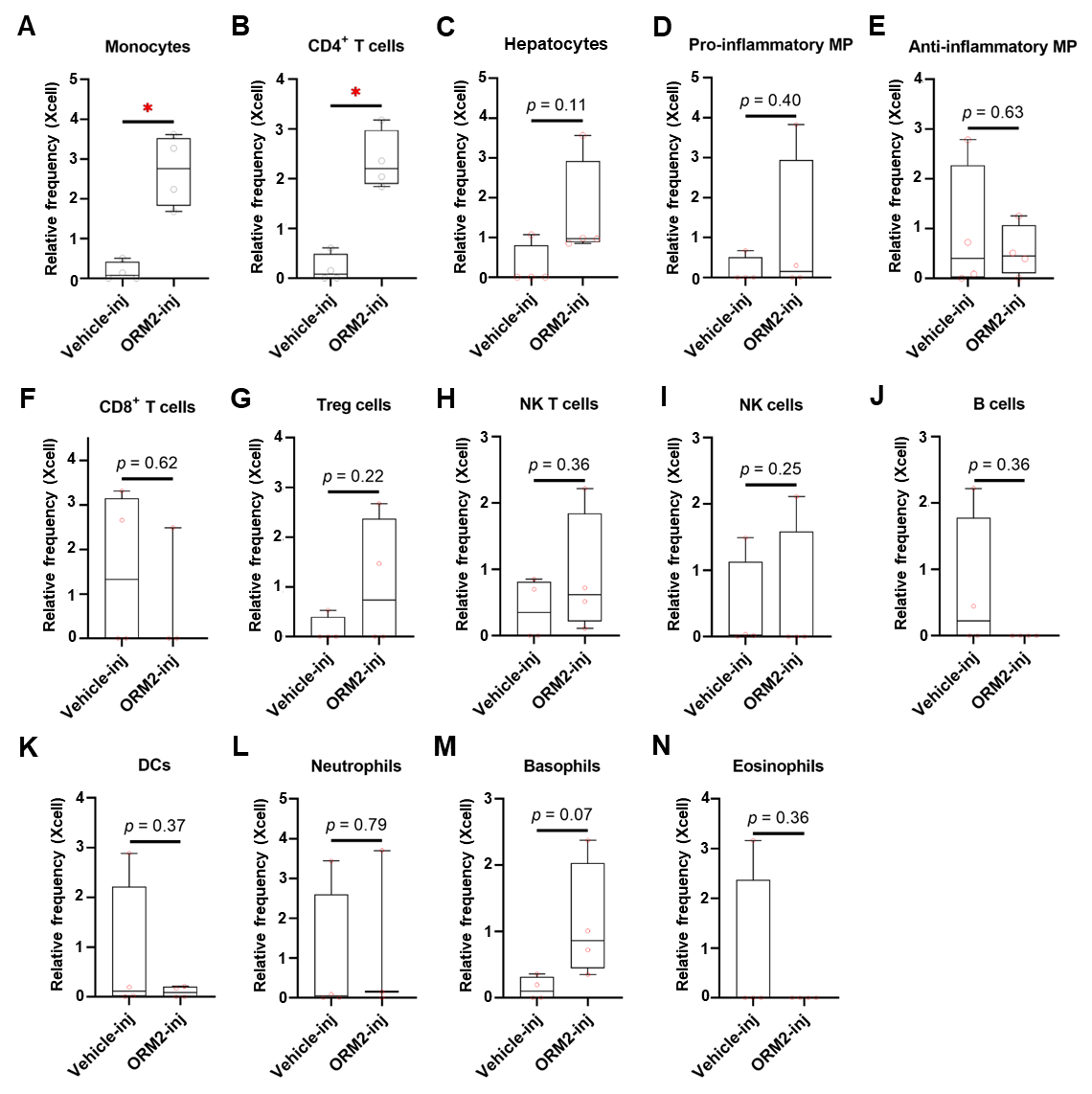


**Figure S2.** The frequency of monocytes and CD4^+^ T cells in mouse livers was increased after ORM2 injection. The bulk RNA-seq data (accession no. GSE186024) generated from healthy WT mouse livers administered with ORM2 was retrieved from the GEO database. Cell frequencies of **(A)** monocytes, **(B)** CD4^+^ T cells, **(C)** hepatocytes, **(D)** pro-inflammatory MP, **(E)** anti-inflammatory MP, **(F)** CD8^+^ T cells, **(G)** Treg cells, **(H)** NK T cells, **(I)** NK cells, **(J)** B cells, **(K)** DCs, **(L)** neutrophils, **(M)** basophils and **(N)** eosinophils in mouse liver were predicted utilizing the Xcell algorithm. Abbreviations: GEO: Gene Expression Omnibus database; NK: natural killer; DC: dendritic cell; Inj: injection; WT: wild type. Unpaired student’s t-tests were performed. **p*<0.05 as indicated or as compared to controls.

.


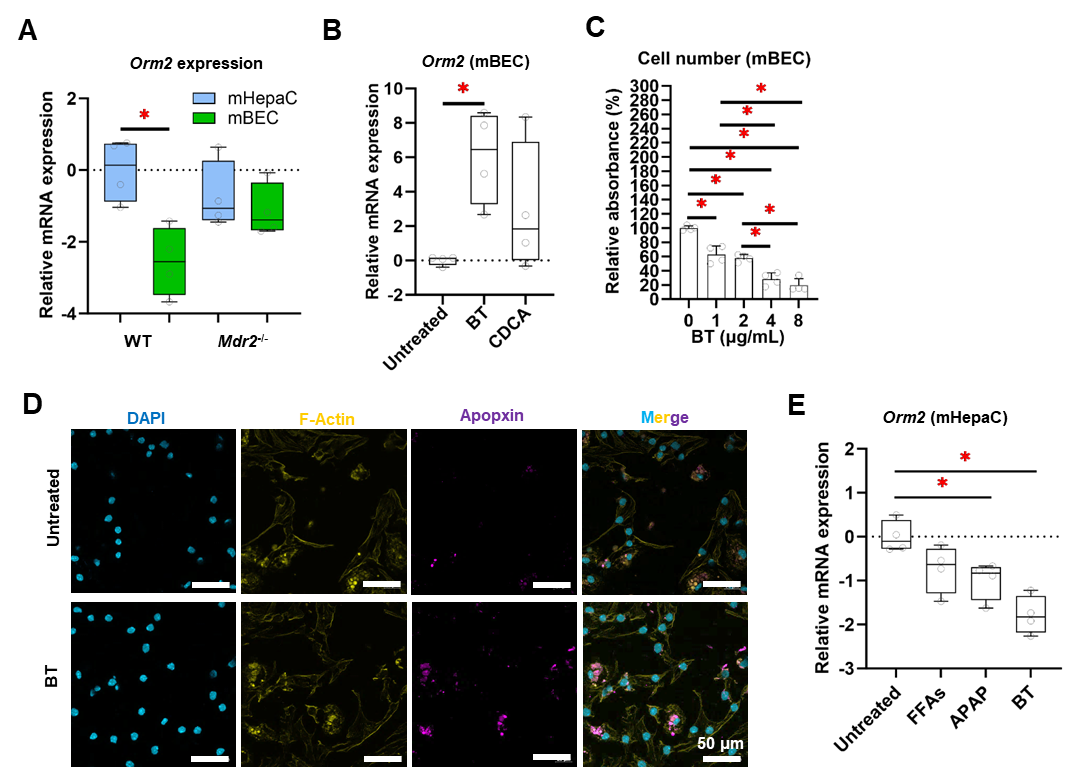


**Figure S3.** *Orm2* expression detected in injured mouse hepatocytes and BECs. **(A)** Gene expression of *Orm2* in WT and *Mdr2*^-/-^ mouse hepatocytes and BECs was measured. **(B)** Gene expression of *Orm2* in mBECs treated with BT or CDCA. **(C)** Cell numbers (relative absorbance) of WT mouse primary BECs treated by BT (0, 1, 2, 4 and 8 μg/mL) for 24 h were elucidated. **(D)** Cell apoptosis (Apopxin^+^) of WT mouse primary BECs was evaluated (BT *vs.* Untreated). **(E)** Gene expression of *Orm2* in mHepaCs treated with FFAs, APAP or BT. Abbreviations: (m)BEC: (mouse) biliary epithelial cell; BT: biliatresone; (m)HepaC: (mouse) hepatocyte; FFAs: free fatty acids; APAP: acetaminophen; WT: wild type. One-way ANOVA followed by Tukey’s multiple comparison test **(B, C and E)** and unpaired student’s t-tests **(B)** were performed. Sample sizes: n = 4 per group. Abbreviations: APAP: acetaminophen; (m)BEC: (mouse) biliary epithelial cell; BT: biliatresone; CDCA: chenodeoxycholic acid; FFAs: free fatty acids; (m)HepaC: (mouse) hepatocyte; HFD: high-fat diet. **p*<0.05 as indicated or as compared to controls.


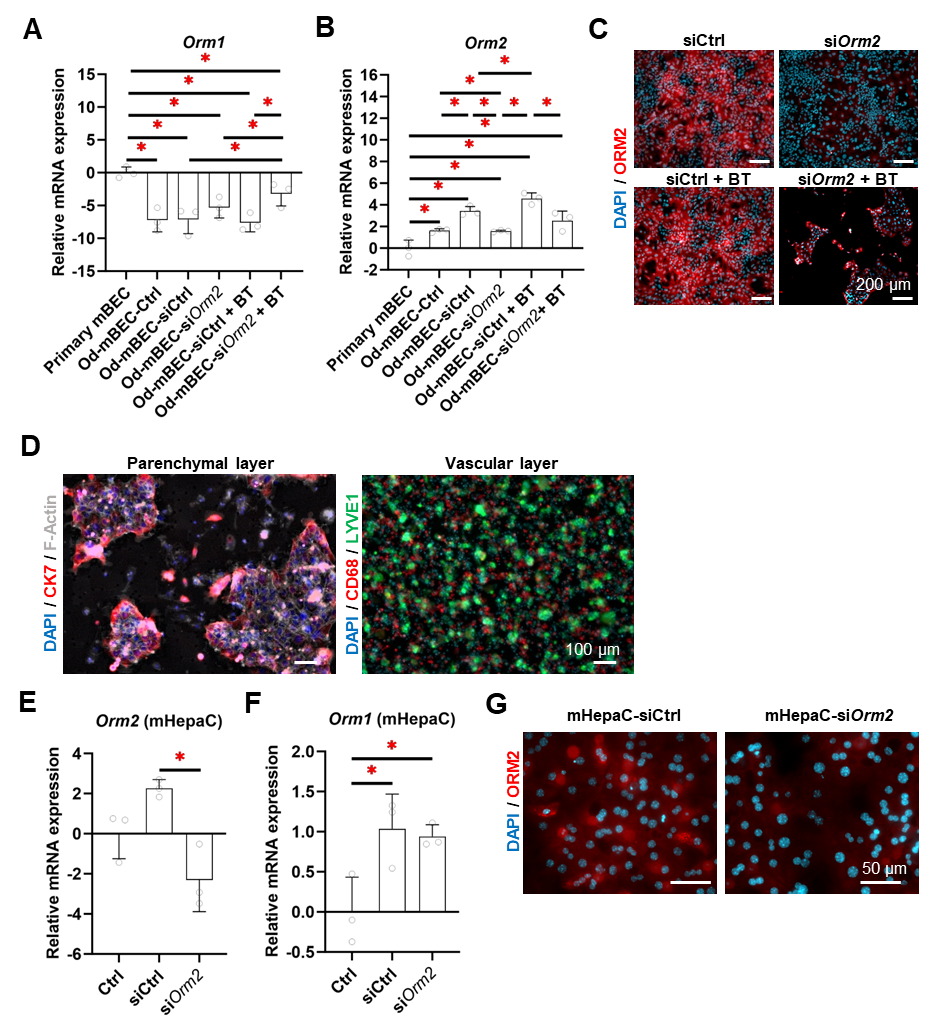


**Figure S4.** *Orm2* expression interference in Od-BECs and HepaCs. Gene expression of **(A)** *Orm1* and **(B)** *Orm2* in WT primary and *Mdr2*^-/-^ mouse Od-BECs upon siCtrl and si*Orm2* along with BT treatment. **(C)** Protein expression of ORM2 in *Mdr2*^-/-^ mouse Od-BECs upon siCtrl and si*Orm2* along with BT treatment was illustrated in fluorescent staining. **(D)** *Mdr2*^-/-^ mouse Od-BECs (CK7^+^F-Actin^+^) and WT mouse primary HSCs (CK7^-^F-Actin^+^) from the parenchymal layer as well as WT mouse KCs (CD68^+^) and LSECs (LYVE1^+^) from the vascular layer were illustrated in fluorescent staining. Gene expression of **(E)** *Orm1* and **(F)** *Orm2* in WT primary hepatocytes upon siCtrl and si*Orm2*. **(G)** Protein expression of ORM2 in WT primary hepatocytes upon siCtrl and si*Orm2* was illustrated in fluorescent staining. Sample sizes: n = 4 per group. Abbreviations: BoC: biliary niche-on-a-chip; Od-(m)BEC: organoid-derived (mouse) biliary epithelial cell; BT: biliatresone; CK7: cytokeratin 7; CD68: cluster of differentiation 68; LoC: liver-on-a-chip; (m)HepaC: (mouse) hepatocytes; WT: wild type. One-way ANOVA followed by Tukey’s multiple comparison tests was performed. **p*<0.05 as indicated or as compared to controls.


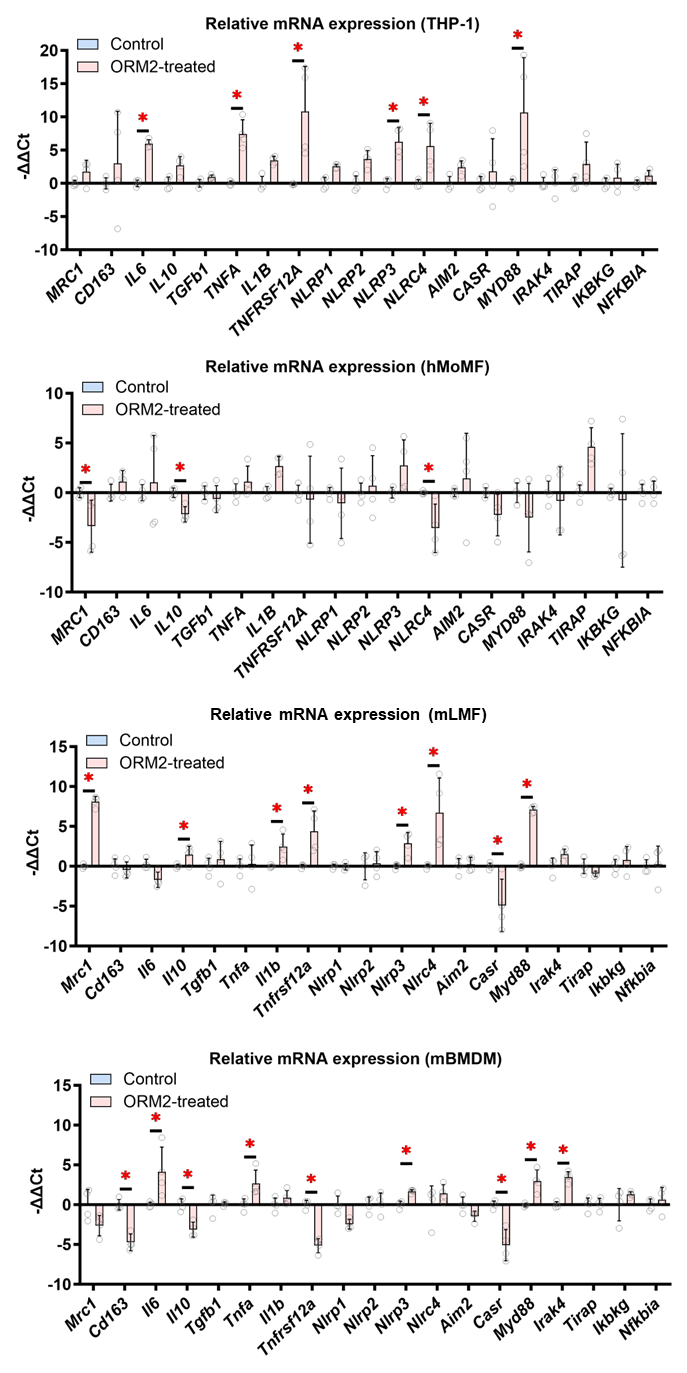


**Figure S5.** Gene expression of macrophage phenotype-associated markers in human and mouse macrophages treated by ORM2 was measured by qRT-PCR method. Unpaired t-tests were performed. Sample sizes: n = 4 per group. Abbreviations: hMoMF: human monocyte-derived macrophages; mBMDM: mouse bone marrow-derived macrophages; mLMF: mouse liver macrophages. **p*<0.05 as indicated or as compared to controls.


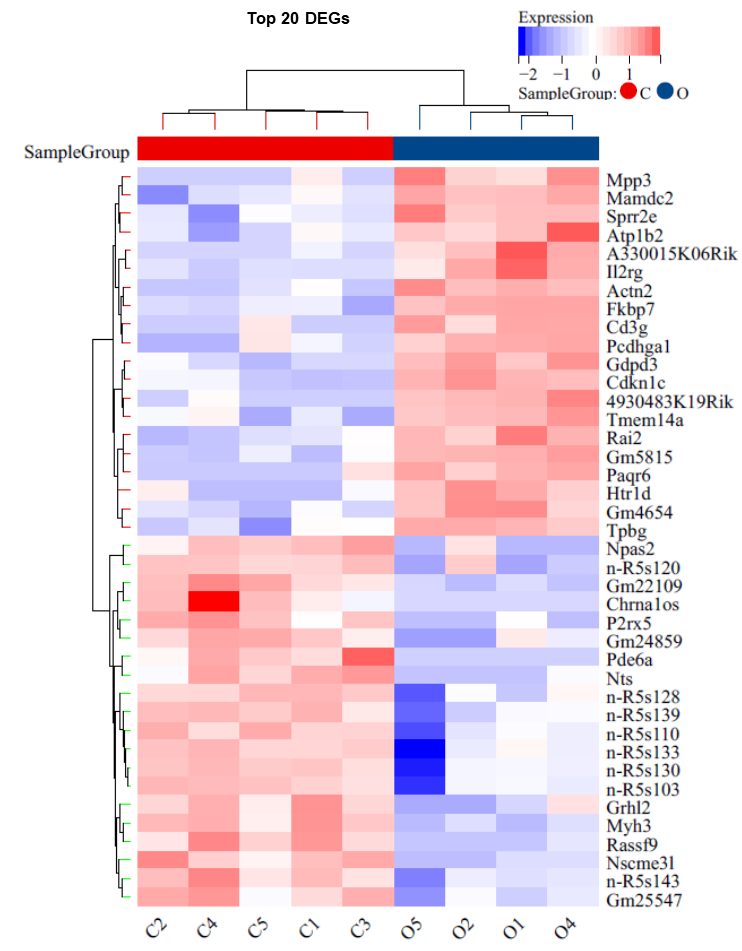


**Figure S6.** Top-20 significantly up- and down-regulated DEGs (*p* < 0.05) generated from bulk RNA-seq data were illustrated in a heatmap [ORM2-treated (n = 5) *vs.* Ctrl (n = 4)].


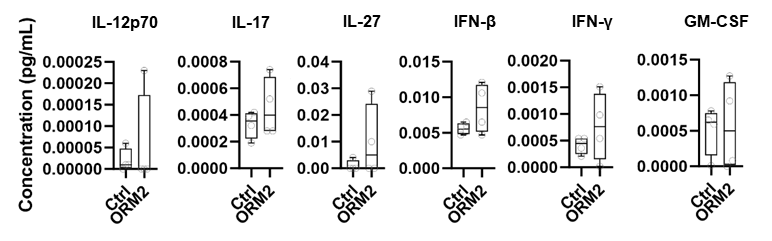


**Figure S7.** Concentrations of IL-12p70, IL-17, IL-27, IFN-β, IFN-γ and GM-CSF in mouse liver macrophage culture were measured. Sample sizes: n = 4 per group. Unpaired t-tests were performed.


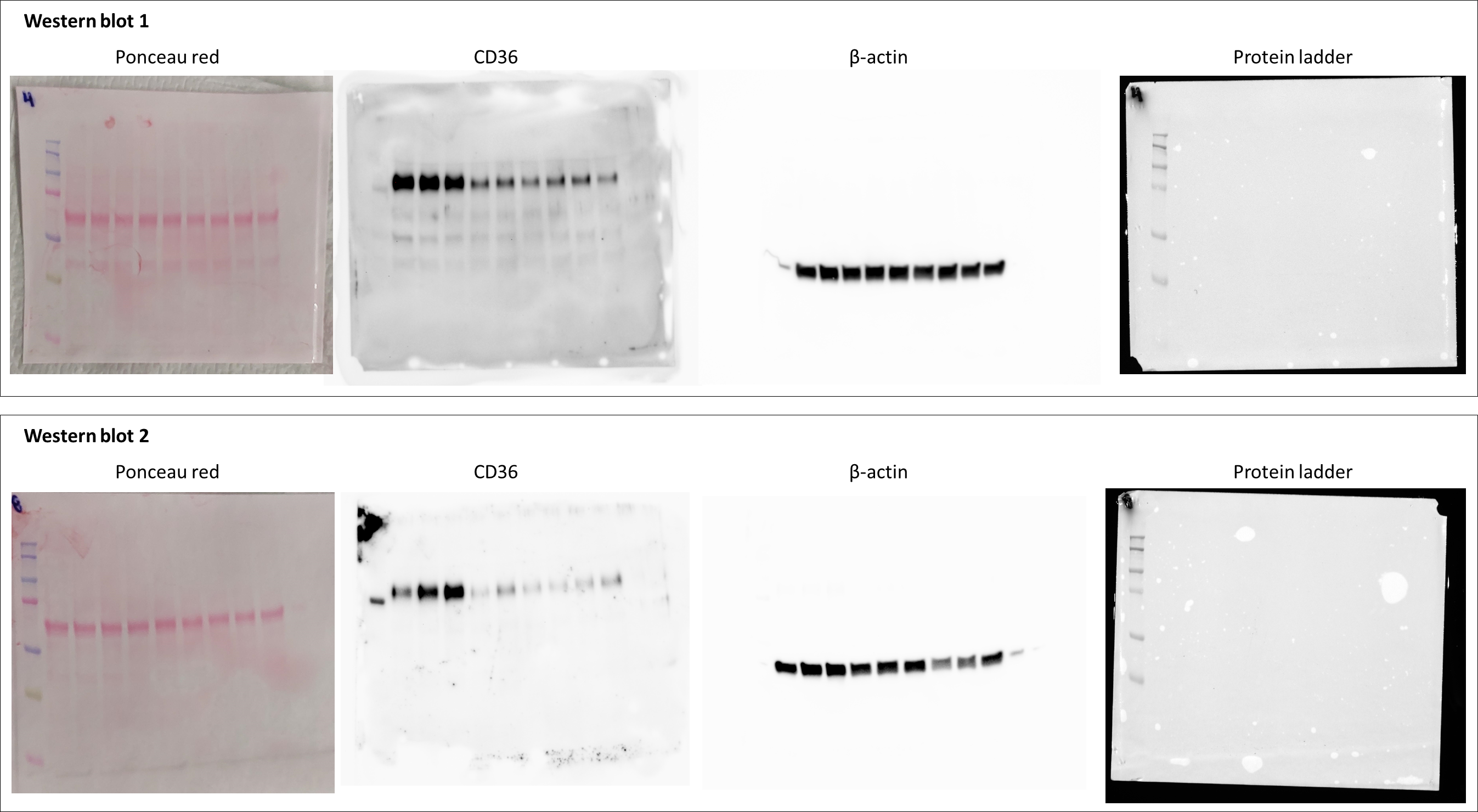


**Figure S8.** Raw whole membrane images from the Western blot experiments. The ponceau red protein labelling, raw bands of CD36 and β-actin, and protein weight indicators from the indicated membranes/experiments were displayed.


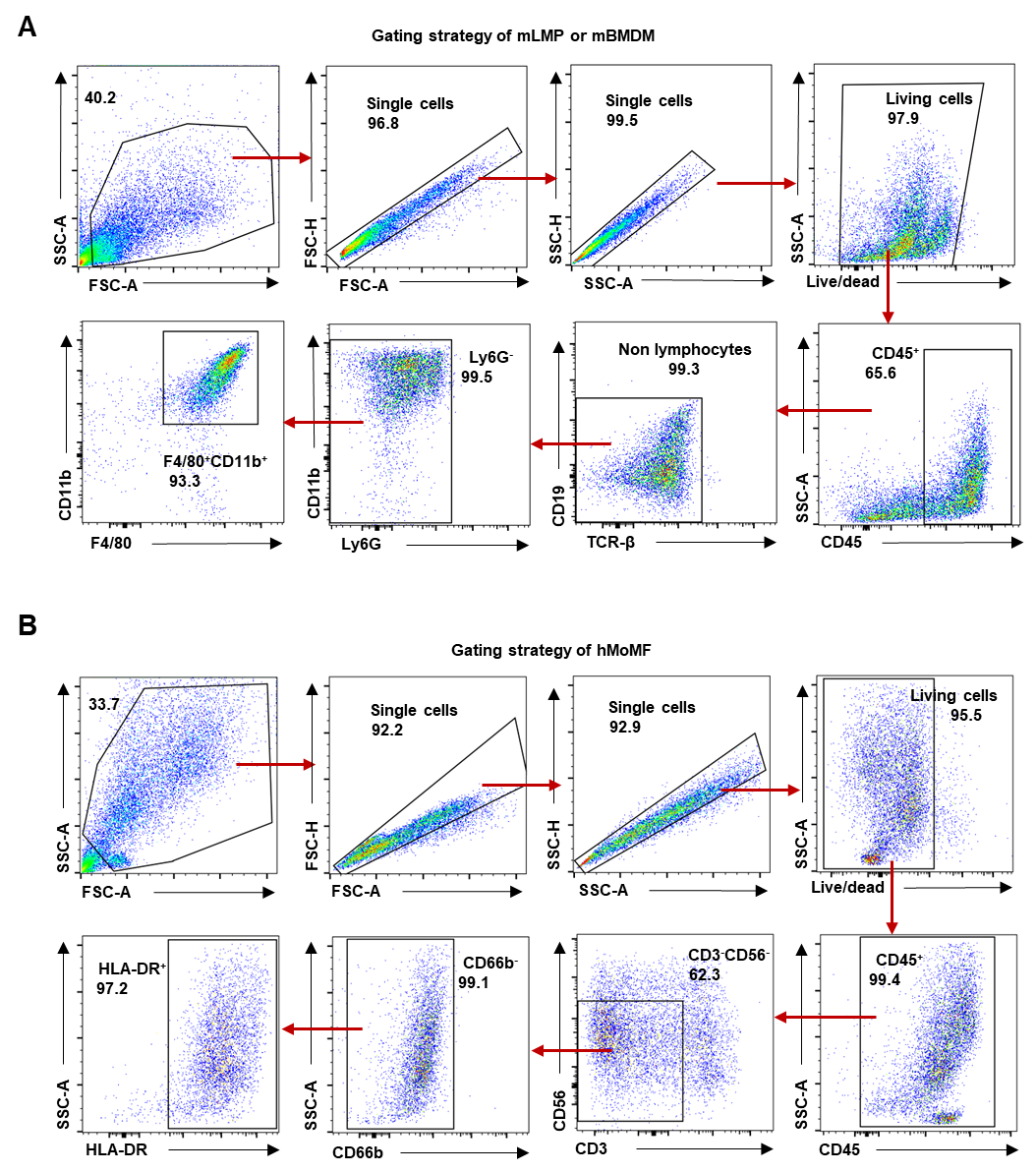


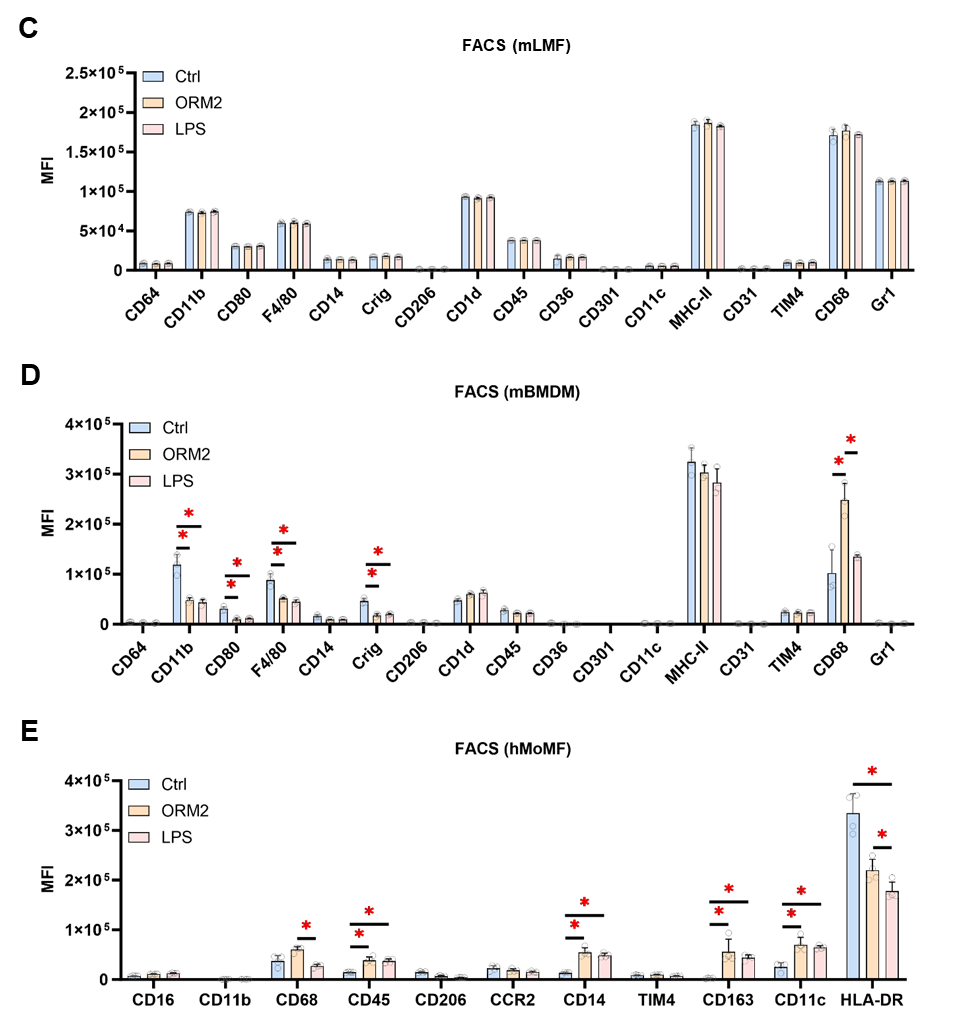


**Figure S9.** Flow cytometry characterized cell phenotypes of mouse liver macrophages, bone marrow-derived macrophages and human monocyte-derived macrophages. Gating strategies of flow cytometry characterization on **(A)** human and **(B)** mouse cells. MFI of diverse macrophage markers in **(C)** mouse liver macrophages, **(D)** bone marrow-derived macrophages and **(E)** human monocyte-derived macrophages. Sample sizes: n = 4 per group. Abbreviations: mLMF: mouse liver macrophages; mBMDM: mouse bone marrow-derived macrophages; hMoMF: human monocyte-derived macrophages. One-way ANOVA followed by Tukey’s multiple comparison tests were performed. **p*<0.05 as indicated or as compared to controls.


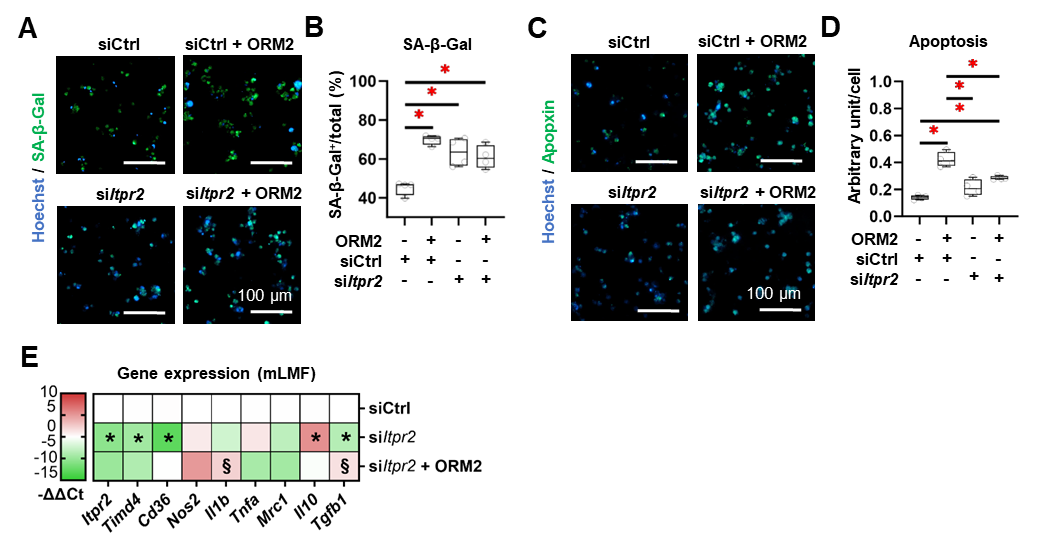


**Figure S10.** ITPR2 suppression ameliorates ORM2-induced cell stress in mouse liver macrophages. **(A)** DNA damage (SA-β-Gal^+^) of mLMF upon siCtrl and si*Itpr2* along with ORM2 treatment was illustrated in fluorescent staining and **(B)** quantitative analysis. **(C)** Cell apoptosis (Apopxin+) of mLMF upon siCtrl and si*Itpr2* along with ORM2 treatment was illustrated in fluorescent staining and **(D)** quantitative analysis. **(E)** Gene expression of *Itpr2*, *Timd4*, *Cd36*, *Nos2*, *Il1b*, *Tnfa*, *Mrc1*, *Il10* and *Tgfb1* in mLMF upon siCtrl and si*Itpr2* along with ORM2 treatment was measured. Sample sizes: n = 4 per group. Abbreviations: mLMF: mouse liver macrophages; SA-β-Gal: Senescence-associated beta-galactosidase. One-way ANOVA followed by Tukey’s multiple comparison tests were performed. ‘*’ represents statistical significance in comparison between si*Itpr2* *vs.* siCtrl. ‘§’ represents statistical significance between si*Itpr2* + ORM2 *vs.* si*Itpr2*. **p*<0.05 as indicated or as compared to controls.

.


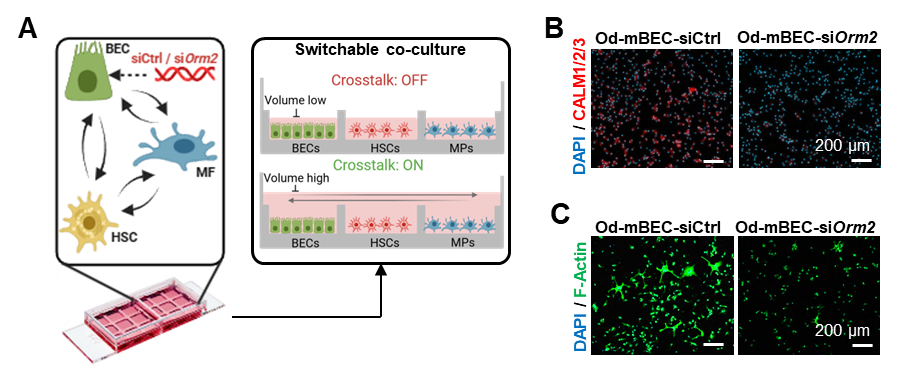


**Figure S11.** Cholangiocyte-derived ORM2 enhances CALM1/2/3 expression in liver macrophages and HSC cytoplasm expansion. **(A)** The scheme of experimental strategy in the co-culture chamber system is illustrated. Mouse primary BECs (transfected with siCtrl or si*Orm2*), MP and HSCs were cultivated in separated chambers. Later, the elevation of culture medium allowed for signal interaction (e.g., cytokines, chemicals) among chambers. **(B)** Protein expression of CALM1/2/3 in WT mouse liver macrophages and **(C)** expansion (F-Actin^+^) of HSCs were investigated by fluorescent staining. Sample sizes: n = 3 per group. Abbreviations: mBEC: mouse biliary epithelial cell; MF: macrophage; HSC: hepatic stellate cell; Od: organoid-derived; WT: wild type.


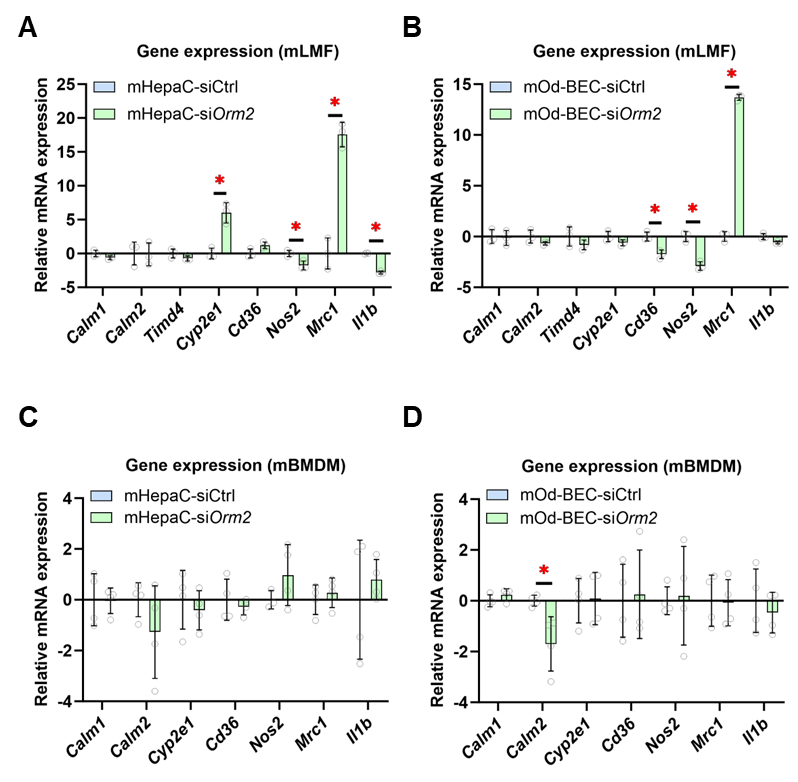


**Figure S12.** Influences of hepatocyte- and Od-BEC-derived ORM2 on mouse liver macrophages and bone marrow derived macrophages. Expression of *Calm1*, *Calm2*, *Timd4*, *Cyp2e1*, *Cd36*, *Nos2*, *Mrc1* and *Il1b* in mLMF treated with conditioned medium from siCtrl or si*Orm2* transfected **(A)** mHepaC and **(B)** mBEC. Expression of *Calm1*, *Calm2*, *Cyp2e1*, *Cd36*, *Nos2*, *Mrc1* and *Il1b* in mBMDM treated with conditioned medium from siCtrl or si*Orm2* transfected **(C)** mHepaC and **(D)** mBEC. Sample sizes: n = 4 per group. Abbreviations: Od: organoid-derived; (m)HepaC: (mouse) hepatocytes (-derived conditioned medium); m(BEC): (mouse) biliary epithelial cells (-derived conditioned medium); mLMF: mouse liver macrophages. mBMDM: bone marrow derived macrophages. Unpaired t-tests were performed. **p*<0.05 as indicated or as compared to controls.


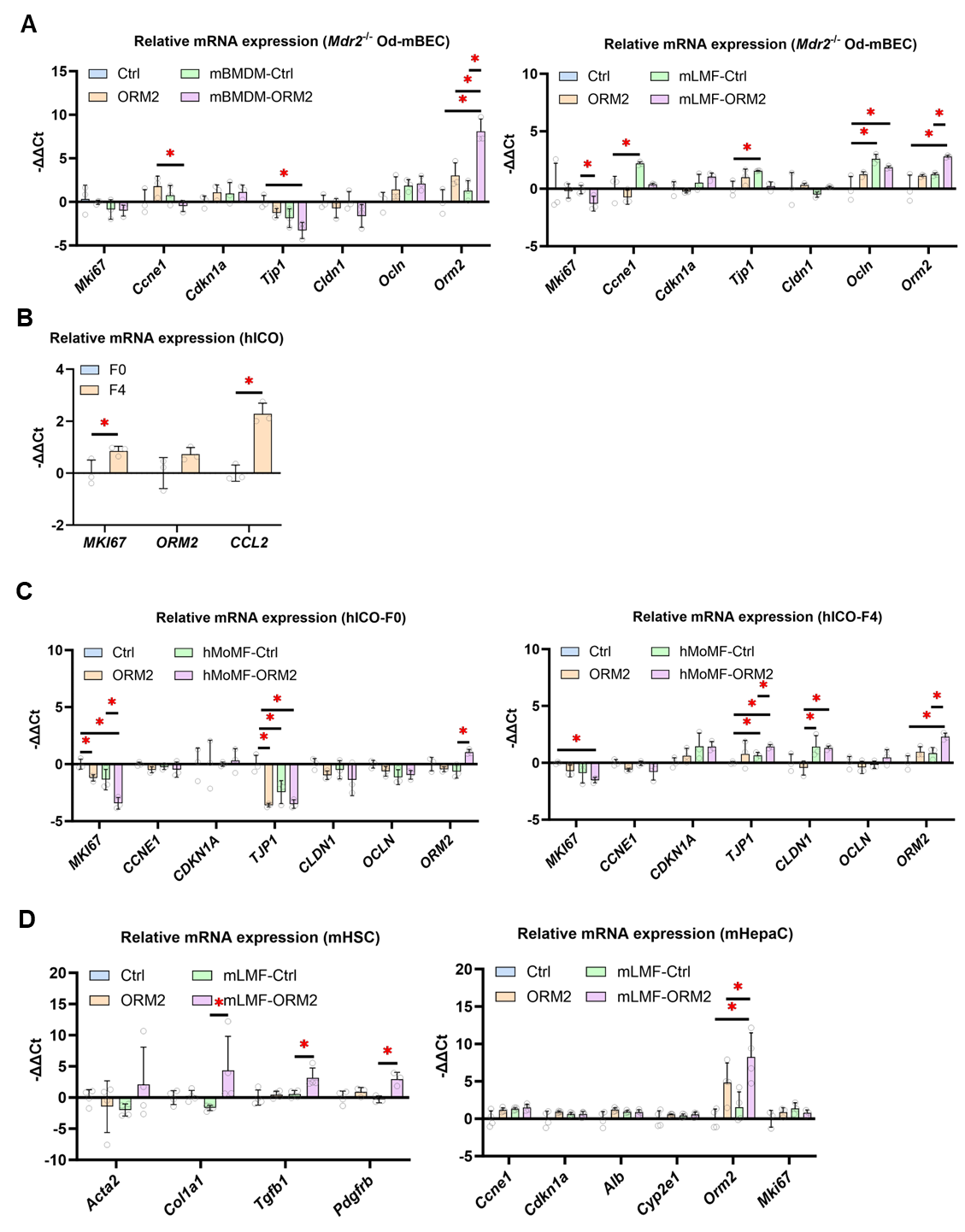


**Figure. S13**. Data points for gene expression in diverse cell experiments. **(A)** Gene expression of *Mki67, Ccne1, Cdkm1a, Tjp1, Cldn1, Ocln* and *Orm2* in *Mdr2*^-/-^ Od-mBECs upon ORM2, mLMF-Ctrl or mLMF-ORM2 treatment. **(B)** Gene expression of *MKI67, ORM2* and *CCL2* in hICOs (F4 vs. F2). **(C)** Gene expression of *MKI67, CCNE1, CDKN1A, TJP1, CLDN1, OCLN* and *ORM2* in hICOs upon ORM2, hMoMF-Ctrl or hMoMF-ORM2 treatment. **(D)** Gene expression of *Acta2*, *Col1a1*, *Tgfb1* and *Pdgfrb* in mHSCs and *Ccne1*, *Cdkn1a*, *Alb*, *Cyp2e1*, *Orm2* and *Mki67* in mHepaCs upon ORM2, mLMF-Ctrl or mLMF-ORM2 treatment. Sample sizes: n = 4 (technical replicates) per group. Abbreviations: hICO(-F0/F4): human intrahepatic cholangiocyte organoids (-Fibrosis stage 1/4); Od: organoid-derived; hMoMF: human monocyte-derived macrophages; mHepaC: mouse hepatocytes; mBEC: mouse biliary epithelial cells; mBMDM: mouse bone marrow-derived macrophages (-derived conditioned medium); mLMF: mouse liver macrophages (-derived conditioned medium). One-way ANOVA followed by Tukey’s multiple comparison tests were performed. **p*<0.05 as indicated or as compared to controls.

**Supplementary Tables**

**Supplementary Table 1. Antibodies/magnetic beads/LEGENDplex beads**

| **Primary antibodies for IHC/ICC/Western blot** | | | | | |
| --- | --- | --- | --- | --- | --- |
| **Name** | **Manufacturer** | **Catalog No.** | **Antibody clone** | **Clone** | **Host species** |
| HRP Anti-beta Actin | Abcam | ab49900 | Monoclonal | AC-15 | Mouse |
| Anti-alpha SMA | Abcam | ab124964 | Monoclonal | ERP5368 | Rabbit |
| Anti-CYP450-2E1 | Abcam | ab28146 | Polyclonal | - | Rabbit |
| Anti-Cytokeratin-19 | DSHB | TROMA-III | Monoclonal | RIgG2a | Rat |
| Anti-CD68 | Abcam | ab237968 | Monoclonal | FA-11 | Rat |
| Anti-Desmin | Abcam | Ab15200 | Polyclonal |  | Rabbit |
| Anti-LYVE1 | Abcam | ab14917 | Polyclonal | - | Rabbit |
| Anti-Collagen-I | Abcam | ab270993 | Monoclonal | EPR24331-53 | Rabbit |
| Anti-CD3 | Abcam | ab16669 | Monoclonal | SP7 | Rabbit |
| Anti-CD11b | Abcam | ab133357 | Monoclonal | EPR1344 | Rabbit |
| Anti-CD45R (B220) | BioLegend | 103202 | Monoclonal | RA3-6B2 | Rat |
| Anti-CLEC4F | R&D | MAB2784 | Monoclonal | 370901 | Rat |
| Anti-Cytokeratin-7 | Agilent | M701829-2 | Monoclonal | OV-TL 12/30 | Mouse |
| Anti-Cytokeratin-7 | Abcam | ab181598 | Monoclonal | EPR17078 | Rabbit |
| Anti-IBA1 | VWR | 100369-764 | Polyclonal | - | Rabbit |
| Anti-MPO | Abcam | ab208670 | Monoclonal | EPR20257 | Rabbit |
| Anti-PCNA | Abcam | ab29 | Monoclonal | PC10 | Mouse |
| Anti-PDGF-Rβ | Abcam | ab32570 | Monoclonal | Y92 | Rabbit |
| Anti-ORM2 | Thermofisher | PA5-119322 | Polyclonal | - | Rabbit |
| Anti-ITPR2 | ABclonal | A19320 | Polyclonal | - | Rabbit |
| Anti-ITPR2 | NOVUS | NB100-2466 | Polyclonal |  | Goat |
| Anti-CALM1/2/3 | ABclonal | A1185 | Polyclonal | - | Rabbit |
| Anti-CD36 | Cell Signaling Technology | 28109 | Monoclonal | E8B7S | Rabbit |
| Anti-TIM4 | Abcam | ab47637 | Polyclonal | - | Rabbit |
| Anti-Ki67 | Abcam | ab16667 | Monoclonal | SP6 | Rabbit |

| **Secondary antibodies** | | | |
| --- | --- | --- | --- |
| **Name** | **Manufacturer** | **Catalog No.** | **Host species** |
| Mouse IgG, Alexa Fluor 555 | Cell Signaling | 4409S | Goat |
| Mouse IgG, Alexa Fluor 647 | Cell Signaling | 4410S | Goat |
| Rabbit IgG, Alexa Fluor 647 | Cell Signaling | 4414S | Goat |
| Rabbit IgG, Alexa Fluor 750 | ThermoFisher | A-21039 | Goat |
| Rat IgG, Alexa Fluor 488 | Cell Signaling | 4416S | Goat |
| Rat IgG, Alexa Fluor 647 | Cell Signaling | 4418S | Goat |
| Peroxidase AffiniPure™ Goat Anti-Rabbit IgG (H+L) | Jackson immuno research | 111-035-003 | Goat |

| **Name** | **Manufacturer** | **Catalog No.** | **Clone** | **Host species** |
| --- | --- | --- | --- | --- |
| CD11b Monoclonal Antibody (M1/70), eFluor™ 450 | Invitrogen | 48-0112-82 | M1/70 | Rat |
| Brilliant Violet 421™ anti-mouse CD4 Antibody | BioLegend | 100437 | GK1.5 | Rat |
| Brilliant Violet 711™ anti-mouse CD8a Antibody | BioLegend | 100747 | 53-6.7 | Rat |
| FITC anti-mouse Ly-6G Antibody | BioLegend | 127605 | 1A8 | Rat |
| CD45 Monoclonal Antibody (30-F11), Alexa Fluor™ 532 | Invitrogen | 58-0451-82 | 30-F11 | Rat |
| PE anti-mouse CD115 (CSF-1R) Antibody | BioLegend | 165003 | W19330C | Rat |
| PE/Cyanine5 anti-mouse CD19 Antibody | BioLegend | 115509 | 6D5 | Rat |
| PE/Cyanine7 anti-mouse TCR β chain Antibody | BioLegend | 109221 | H57-597 | Rat |
| Alexa Fluor® 647 anti-mouse F4/80 Antibody | BioLegend | 123122 | BM8 | Rat |
| Alexa Fluor® 700 anti-mouse CD11c Antibody | BioLegend | 117319 | N418 | Rat |
| APC/Cyanine7 anti-mouse NK-1.1 Antibody | BioLegend | 108723 | PK136 | Rat |
| APC/Fire™ 810 anti-mouse Ly-6G/Ly-6C (Gr-1) Antibody | BioLegend | 108469 | RB6-8C5 | Rat |
| PE/Fire™ 810 anti-mouse I-A/I-E Antibody | BioLegend | 107667 | M5/114.15.2 | Rat |
| APC anti-mouse CD192 (CCR2) Antibody | BioLegend | 150627 | SA203G11 | Rat |
| PE Anti-HLA DR + DP + DQ antibody | Abcam | ab23901 | WR18 | Mouse |
| Brilliant Violet 421™ anti-mouse CD64 (FcγRI) Antibody | BioLegend | 139309 | X54-5/7.1 | Mouse |
| Brilliant Violet 605™ anti-mouse CD80 Antibody | BioLegend | 104729 | 16-10A1 | Armenian Hamster |
| Brilliant Violet 650™ anti-mouse F4/80 Antibody | BioLegend | 123149 | BM8 | Rat |
| Brilliant Violet 605™ anti-mouse CX3CR1 Antibody | BioLegend | 149027 | SA011F11 | Mouse |
| BV711 Rat Anti-Mouse CD14 | BD | 740692 | rmC5-3 | Rat |
| BV750 Rat Anti-Mouse CRIg | BD | 749500 | 17C9 | Rat |
| Brilliant Violet 785™ anti-mouse CD206 (MMR) Antibody | BioLegend | 141729 | C068C2 | Rat |
| FITC anti-mouse CD1d (CD1.1, Ly-38) Antibody | BioLegend | 123507 | CD1.1, Ly-38 | Rat |
| PerCP/Cyanine5.5 anti-mouse CD11c Antibody | BioLegend | 117327 | N418 | Armenian Hamster |
| Brilliant Violet 421™ anti-mouse CD64 (FcγRI) Antibody | Biolegend | 139309 | X54-5/7.1 | Mouse |
| PE/Dazzle™ 594 anti-mouse CD301 (MGL1/MGL2) Antibody | BioLegend | 145714 | LOM-14 | Rat |
| PE/Cyanine7 anti-mouse Tim-4 Antibody | BioLegend | 130010 | RMT4-54 | Rat |
| APC anti-mouse CD163 Antibody | BioLegend | 155306 | S15049I | Rat |
| Alexa Fluor® 700 anti-mouse Ly-6G Antibody | BioLegend | 127622 | 1A8 | Rat |
| APC/Cyanine7 anti-mouse CD68 Antibody | BioLegend | 137023 | FA-11 | Rat |
| APC/Fire™ 810 anti-mouse Ly-6G/Ly-6C (Gr-1) Antibody | BioLegend | 108470 | RB6-8C5 | Rat |
| PE/Dazzle™ 594 anti-human CD192 (CCR2) Antibody | BioLegend | 357221 | K036C2 | Mouse |
| Brilliant Violet 785™ anti-human CD68 Antibody | BioLegend | 333826 | Y1/82A | Mouse |
| Brilliant Violet 570™ anti-human HLA-DR Antibody | BioLegend | 307638 | L243 | Mouse |
| V500 Mouse Anti-Human CD16 | BD | 561394 | 3G8 | Mouse |
| Alexa Fluor® 700 anti-human CD11c Antibody | BioLegend | 337220 | Bu15 | Mouse |
| CD45 Monoclonal Antibody (HI30), Alexa Fluor™ 532 | eBioscience | 58-0459-42 | HI30 | Mouse |
| CD206 (MMR) Monoclonal Antibody (19.2), PE | eBioscience | 12-2069-41 | 19.2 | Mouse |
| BV711 Rat Anti-CD11b | BD | 563168 | M1/70 | Rat |
| CD163 Monoclonal Antibody (MAC 2-158), Alexa Fluor™ 647 | eBioscience | 51-1637-42 | MAC 2-158 | Mouse |
| PerCP/Cyanine5.5 anti-human CD14 Antibody | BioLegend | 301824 | M5E2 | Mouse |
| PE/Cyanine7 anti-human Tim-4 Antibody | BioLegend | 354005 | 9F4 | Mouse |

| **Magnetic beads** | | |
| --- | --- | --- |
| **Name** | **Manufacturer** | **Catalog No.** |
| Mouse anti-F4/80 Microbeads | Milteny | 5210707948 |
| Mouse anti-CD146 Microbeads | Milteny | 5190725032 |
| Mouse anti-CD326 (EpCAM) Microbeads | Milteny | 70285065-00 |
| **LEGENDplex beads** | | |
| **Target** | **Manufacturer** | **Beads ID** |
| IL-23 | BioLegend | A4 |
| IL-1α | BioLegend | A5 |
| IFN-γ | BioLegend | A6 |
| TNF-α | BioLegend | A7 |
| MCP-1 | BioLegend | A8 |
| IL-12p70 | BioLegend | A10 |
| IL-1β | BioLegend | B2 |
| IL-10 | BioLegend | B3 |
| IL-6 | BioLegend | B4 |
| IL-27 | BioLegend | B5 |
| IL-17A | BioLegend | B6 |
| IFN-β | BioLegend | B7 |
| GM-CSF | BioLegend | B9 |

**Supplementary Table 2. Primer sequences used for gene expression analyses**

**Primer sequences for mouse cell analyses**

| **Genes** | **Forward 5'-3'** | **Reverse 5'-3'** |  |
| --- | --- | --- | --- |
| *18S* | AACTTTCGATGGTAGTCGCCGT | TCCTTGGATGTGGTAGCCGTTT |  |
| *Acta2* | TGACAGAGGCACCACTGAACC | TCCAGAGTCCAGCACAATACCAGT |  |
| *Aim2* | CTGCCGCCATGCTTCCTTA | AGTCCCAGGATCAGCCTAGA |  |
| *Calm1* | TATATATCGCGGCACACAGGC | ATGGTGCCATTGCCATCAGC |  |
| *Calm2* | CCCTTGCAGCATGAGTTCAAA | ACGCAGAGTTACAGCTCCAC |  |
| *Casr* | TGCCTTGTGATCCTCTTTCCAT | TCCACGGAAGTTATACCTGATG |  |
| *Ccne1* | CTCCCACAACATCCAGACCC | AGCAACCTACAACACCCGAG |  |
| *Ccl2* | GTG TTG GCT CAG CCA GAT GC | GACACCTGCTGCTGGTGATCC |  |
| *Ccl3* | ACCATGACACTCTGCAACCAAG | TCTGCCGGTTTCTCTTAGTCAGG |  |
| *Ccl5* | CTGCTGCTTTGCCTACCTCTCC | GGCACACACTTGGCGGTTCC |  |
| *Ccr2* | TCGCTGTAGGAATGAGAAGAAGAGG | CAAGGATTCCTGGAAGGTGGTCAA |  |
| *Cd163* | GTGCTGGATCTCCTGGTTGT | CGTTAGTGACAGCAGAGGCA |  |
| *Cd36* | TGAATGGTTGAGACCCCGTG | CGTGGCCCGGTTCTACTAAT |  |
| *Cldn1* | TGGGGCTGATCGCAATCTTT | CACTAATGTCGCCAGACCTGA |  |
| *Col1a1* | TCTGACTGGAAGAGCGGAGAG | GGCACAGACGGCTGAGTAGG |  |
| *Cxcl5* | TCCTCAGTCATAGCCGCAAC | GCTTTCTTTTTGTCACTGCCC |  |
| *Cyp2e1* | ATAGAAGTTGGAACCTGCCC | CTTTGCCAACTTGGTTAAAGAC |  |
| *Fgf1* | TTATACGGCTCGCAGACAC | TGCTTCTTGGAGGTGTAAGTG |  |
| *Fgf2* | CGACCCACACGTCAAACTAC | GCACACACTCCCTTGATAGAC |  |
| *Icam1* | CATCACCGTGTATTCGTTTC | GTGAGGTCCTTGCCTACTTG |  |
| *Ifng* | GGAGGAACTGGCAAAAGGATGG | TGTTGCTGATGGCCTGATTGTC |  |
| *Ikbkg* | CTTGTTTTGGCTCAGCCTGC | GTCCTCAGCCATCTGCTGTT |  |
| *Il10* | GGCTGAGGCGCTGTCATCG | TCATTCATGGCCTTGTAGACACC |  |
| *Il1a* | CGCTTGAGTCGGCAAAGAAAT | AAGGTGCTGATCTGGGTTGG |  |
| *Il1b* | GAGCTGAAAGCTCTCCACCTC | CTTTCCTTTGAGGCCCAAGGC |  |
| *Il23* | ACCAGCGGGACATATGAATCT | AGACCTTGGCGGATCCTTTG |  |
| *Il6* | GCTACCAAACTGGATATAATCAGGA | CCAGGTAGCTATGGTACTCCAGAA |  |
| *Irak4* | CCCAAACCGTCAAAAGCCTG | GTTCTCGTGCTGACACGTTG |  |
| *Itpr2* | CGAGGGTGATAATGTGAATGCTG | AGGATCCCAAACACCTGTGC |  |
| *Mki67* | ACCATCATTGACCGCTCCTT | TTGACCTTCCCCATCAGGGT |  |
| *Mrc1* | TTCCGCTGGGTGTCAGATTC | TCTCGCTTCCCTCAAAGTGC |  |
| *Myd88* | ATGACCCCCTAGGACAAACG | GAGAATCTGGCTCCGCATCA |  |
| *Nfkbia* | TGTGATCACCAACCAGCCAG | AGACACGTGTGGCCATTGTAG |  |
| *Nlrc4* | GGATTGCTTGGCCAGGAGAG | CAGGTCTTCTTCTGTGACCTGA |  |
| *Nlrp1* | ATAGAGGAGCAGGCAGGTCT | CGTGCTCCTGGAAAGGTTCT |  |
| *Nlrp2* | GGCCTCCTGAATGAGACTCG | TTGTTCATCCGGGGACCTTT |  |
| *Nlrp3* | TGGGTTCTGGTCAGACACGAG | GGGGCTTAGGTCCACACAGAAA |  |
| *Nos2* | GCCCAGCCAGCCCAAC | GCACATCAAAGCGGCCATAG |  |
| *Olcn* | TTGAACTGTGGATTGGCAGC | CAAGATAAGCGAACCTTGGCG |  |
| *Orm1* | AGTGCTGAGGAAACATGGGG | GCTGACCGCACCTATCCTTT |  |
| *Orm2* | TTGGTGCGGCTGTCCTAAA | GCTGACTGCACCTGTCCTTTT |  |
| *Cdkn1a/P21* | AGAATAAAAGGTGCCACAGGC | AATCTGTCAGGCTGGTCTGC |  |
| *Pdgfrb* | CATGTCTGAGACCCGGTACG | AGTCGTAAGGCAACTGCACA |  |
| *Pecam1* | GAAGGTGCATGGCGTATC | TTCTTGCAAGGAACAATTGAC |  |
| *Spp1* | AAGAAGCATCCTTGCTTGGGT | GGTCGTAGTTAGTCCTTGGCT |  |
| *Tgfb1* | GCTCGCTTTGTACAACAGCACC | GCGGTCCACCATTAGCACG |  |
| *Timd4* | AGAGACACAAGAGGCCAGACA | TAGGGGCTGGAGGCTTATTCC |  |
| *Tnfa* | ACCACGCTCTTCTGTCTACTGA | TCCACTTGGTGGTTTGCTACG |  |
| *Tnfrsf12a* | GCCTCGAAGAAGTGCTCCTAAA | CCTTAAGATGAGCCCAGGGGA |  |
| *Tjp1* | GAGCAGGCTTTGGAGGAGAC | CATTGCTGTGCTCTTAGCGG |  |
| *Tirap* | ACCTGCATCCAGAACAGTAAGT | GGAGGGCATTTGAGATCCGT |  |
| *Vcam1* | TGTTTGCAGTCTCTCAAGC | GGCTGTCTATCTGGGTTCTC |  |
| **Primer sequences for human cell analyses** | | |  |
| **Genes** | | **Forward 5'-3'** | **Reverse 5'-3'** |
| *ACTB* | | GATTCCTATGTGGGCGACGA | CACAGGACTCCATGCCCAG |
| *AIM2* | | CCCAGGGATCAGGAGTTGATAAG | GACTTTTGGTGCAGCACGTT |
| *CASR* | | GGGAGCCACTCACCTTTGTG | AGGCACTGGCATCTGTCTCA |
| *CD163* | | AAAAAGCCACAACAGGTCGC | GGTATCTTAAAGGCTCACTGGGT |
| *IKBKG* | | GACTCTGCTGACAGCCCTTG | CCTGGCATTCCTTAGTGGCA |
| *IL10* | | GGTTGCCAAGCCTTGTCTGAG | GATGACAGCGCCGTAGCC |
| *IL1B* | | AGCCATGGCAGAAGTACCTG | CCTGGAAGGAGCACTTCATCT |
| *IL6* | | GGCATCTCAGCCCTGAGAAAG | CACCAGGCAAGTCTCCTCATT |
| *IRAK4* | | AAGGCATTCCCCGCCTTAAT | TTTGGGAACAGCATCTGGGA |
| *MRC1* | | TTCCTTTGGACGGATGGACG | GTCAAGGAAGGGTCGGATCG |
| *MYD88* | | CAGCCAGAGGAGAGGAGGAT | GGTTGGTGTAGTCGCAGACA |
| *NFKBIA* | | ATGTCAATGCTCAGGAGCCC | GGTCAGTCACTCGAAGCACA |
| *NLRC4* | | AGGCCTCACTGAAACGGAA | AAACTACTCTTCATTCTGGCTGA |
| *NLRP1* | | TCAAACTCCTGGACGTGAGC | CAGAGCTCCAGTTCCTTCCG |
| *NLRP2* | | AACAGAAGCACAAGACAAAGAC | GTGCTTGGGCTAGGATGTGT |
| *NLRP3* | | CTGGCATCTGGGGAAACCT | CTTAGGCTTCGGTCCACACA |
| *TGFB1* | | GCCCTGGACACCAACTATTGCT | ACGCTCCAAATGTAGGGGCAGG |
| *TNFA* | | GCCCATGTTGTAGCAAACCC | GGAGGTTGACCTTGGTCTGG |
| *TNFRSF12A* | | CCTCGCAGAAGTGCACCTAAA | TCAGGTAGACAGCCTTCCCC |
| *TIRAP* | | GCGCAGGCCTTACATAGGAA | GGAGCAGCCATCAGGGTATG |
